# *ORIGIN OF RECOGNITION COMPLEX 3* controls the development of maternal excess endosperm in the *Paspalum simplex* agamic complex (*Poaceae*)

**DOI:** 10.1093/jxb/erad069

**Published:** 2023-02-22

**Authors:** Michele Bellucci, Maria Eugenia Caceres, Francesco Paolocci, Juan Manuel Vega, Juan Pablo Amelio Ortiz, Marilena Ceccarelli, Francesca De Marchis, Fulvio Pupilli

**Affiliations:** Institute of Biosciences and Bioresources (IBBR), National Research Council (CNR), 06128, Perugia, Italy; Institute of Biosciences and Bioresources (IBBR), National Research Council (CNR), 06128, Perugia, Italy; Institute of Biosciences and Bioresources (IBBR), National Research Council (CNR), 06128, Perugia, Italy; Instituto de Investigaciones en Ciencias Agrarias de Rosario (IICAR), CONICET-UNR and Laboratorio de Biología Molecular, Facultad de Ciencias Agrarias, Universidad Nacional de Rosario, S2125ZAA, Zavalla, Argentina; Instituto de Investigaciones en Ciencias Agrarias de Rosario (IICAR), CONICET-UNR and Laboratorio de Biología Molecular, Facultad de Ciencias Agrarias, Universidad Nacional de Rosario, S2125ZAA, Zavalla, Argentina; Department of Chemistry, Biology and Biotechnology, University of Perugia, 06123, Perugia, Italy; Institute of Biosciences and Bioresources (IBBR), National Research Council (CNR), 06128, Perugia, Italy; Institute of Biosciences and Bioresources (IBBR), National Research Council (CNR), 06128, Perugia, Italy; Ohio State University, USA

**Keywords:** Apomixis, cell cycle, interploidy crosses, maternal excess endosperm, *ORC3*, *Paspalum simplex*

## Abstract

Pseudogamous apomixis in *Paspalum simplex* generates seeds with embryos genetically identical to the mother plant and endosperms deviating from the canonical 2(maternal):1(paternal) parental genome contribution into a maternal excess 4m:1p genome ratio. In *P. simplex*, the gene homologous to that coding for subunit 3 of the *ORIGIN OF RECOGNITION COMPLEX* (*PsORC3*) exists in three isogenic forms: *PsORC3a* is apomixis specific and constitutively expressed in developing endosperm whereas *PsORCb* and *PsORCc* are up-regulated in sexual endosperms and silenced in apomictic ones. This raises the question of how the different arrangement and expression profiles of these three *ORC3* isogenes are linked to seed development in interploidy crosses generating maternal excess endosperms. We demonstrate that down-regulation of *PsORC3b* in sexual tetraploid plants is sufficient to restore seed fertility in interploidy 4*n*×2*n* crosses and, in turn, its expression level at the transition from proliferating to endoreduplication endosperm developmental stages dictates the fate of these seeds. Furthermore, we show that only when being maternally inherited can *PsORC3c* up-regulate *PsORC3b*. Our findings lay the basis for an innovative route—based on *ORC3* manipulation—to introgress the apomictic trait into sexual crops and overcome the fertilization barriers in interploidy crosses.

## Teaser

We provide the first evidence of a gene genetically linked to apomictic reproduction, which is functionally involved in unbalanced endosperm development.

## Introduction

Sexual reproduction creates new allele arrangements via meiotic recombination, segregation, and fertilization, allowing species to rapidly adapt to environmental changes. Unlike animals, where meiotic products directly differentiate into ­gametes, plants generate, by meiotic division, multicellular female and male gametophytes that envelop the gametes. Angiosperm seeds are formed after two fertilization events: first the fusion of the egg cell nucleus (*n*) enclosed in the female gametophyte (the embryo sac) with a sperm nucleus (*n*) of the pollen, which leads to the formation of the zygote (2*n*). The latter then divides to form the embryo, and the fusion of polar nuclei of the embryo sac (*n*+*n*) with a second sperm nucleus (*n*) generates the endosperm (3*n*). Although genetically similar, embryo and endosperm differ in the ploidy level, with the embryo being diploid (2*x*) whereas the endosperm is usually triploid (3*x*).

Besides sexuality, some plants have developed alternative routes of asexual reproduction including variable forms of vegetative propagation and apomixis. The latter, defined as clonal reproduction through seeds (Nogler, 1984), refers to the formation of true seeds whose embryos are genetically identical to the mother plant. The use of apomixis in agriculture relies upon the idea of fixing and propagating superior genotypes (i.e. F_1_ hybrids) through seeds without loss of heterosis. The potential impact of widespread use of apomixis in major crops has been estimated to be analogous to or even greater than the green revolution effect of the 1960s (Hanna, 1995). Among several variants of apomictic reproduction (Pupilli and Barcaccia, 2012), gametophytic aposporous apomixis (hereinafter defined as apomixis) deals with the formation of an unreduced embryo sac through three mitosis cycles of a nucellus differentiated cell (i.e. apospory initial), followed by the development of the egg cell into an embryo by parthenogenesis (without fertilization). Being rarely capable of autonomous development, endosperm more frequently develops after fertilization of one or both unreduced polar nuclei by a reduced sperm nucleus (pseudogamous apomixis). Except for a few forage species and some fruit trees, no apomictic major crop has been identified so far. Past attempts to transfer apomixis from wild apomictic relatives to crops have only yielded agronomically unsuitable biological materials due to chromosome instability and sterility (Vielle-Calzada *et al.*, 1996). Artificial apomictic phenotypes have partially been recreated in sexual model plants such as Arabidopsis (Mohan *et al.*, 2011) and rice (Khanday *et al.*, 2019; Wang *et al.*, 2019) based on the manipulation of sexual pathway genes mimicking specific aspects of natural apomixis. Although these plants represent the first proof-of-principle of possible apomixis reproduction in a sexual background, Arabidopsis mutants cannot be considered as genuinely apomictic because they still rely on crossing to express the trait. In the case of rice, where the engineered apomixis system partially relies on a gene derived from a natural apomictic species (*BABYBOOM1*, see below), the clonal seed production seems to be satisfactory (Vernet *et al.*, 2022). Therefore, the development of an apomixis system suitable to induce a stable apomictic reproduction in crops should be based on a better understanding of gene networks governing the trait in a natural agamic complex (i.e. species including both sexual and apomictic genotypes), whose reproductive phenotypes have already been stabilized under natural selection. In fact, in these species, the gene networks controlling apomixis are probably formed by the genetic determinants of the traits that are specific to the apomictic types (or apomixis-linked), and by downstream-acting genes, whose expression is differently regulated in the two genotypes. Genuine sexual species *per se* might lack, or cannot be naturally committed to express, genes acting downstream of the apomixis-linked factors. In this view, among several apomixis-related genes isolated in natural apomictic systems (Xu *et al.*, 2022), only two apomixis-linked genes have been characterized as definitely genetic determinants of parthenogenesis: *BABY BOOM LIKE* (*BBL*) in *Pennisetum squamulatum* (Conner *et al.*, 2015) and *PARTHENOGENESIS* (*PAR*) (Underwood *et al.*, 2022) in *Taraxacum officinale*.

The grass genus *Paspalum*, as a whole, is one of the most frequently used biological model systems to study natural apomixis (Ortiz et al., 2013, 2020), and genes essential for aposporous embryo sac formation have been identified in *P. notatum* (Siena *et al.*, 2014; Mancini *et al.*, 2018). Furthermore, a gene homologous to the Arabidopsis *PROTEIN DISULFIDE ISOMERASE-Like 2-1* (*PDIL2-1*), putatively involved in endosperm balance number (EBN) insensitivity (see below), has been identified in *P. notatum* and functionally characterized in Arabidopsis (Pozzi *et al.*, 2021). The species *P. simplex* exists in nature as an agamic complex including sexual diploid (2*n*=2*x*=20) and apomictic polyploid (mainly tetraploid, 2*n*=4*x*=40) cytotypes (Caceres *et al.*, 2001). In the sexual type, a single, reduced, eight-nucleated embryo sac develops after three mitotic divisions of the functional megaspore. The mature sexual embryo sac is composed of an egg apparatus (one egg cell and two synergid cells) at the micropylar end, a binucleated central cell, and three or more antipodal cells at the chalazal end (Caceres *et al.*, 2001). In apomictic plants, all four megaspores degenerate after meiosis and only one nucellar cell gives rise, after three mitotic divisions, to an eight-nucleated aposporous embryo sac (which is similar in shape to the meiotic one). In rare cases, both meiotic and aposporous embryo sacs develop together within the same ovule (Caceres *et al.*, 2001). Polar nuclei are fertilized by a reduced sperm nucleus in both sexual and apomictic paths; however, whereas the sexual egg is fertilized by a sperm nucleus, an apomictic egg cell autonomously develops into an embryo by parthenogenesis. Seeds formed through one or the other route of development can be differentiated by the ratio between the relative DNA content (expressed as the C value) in the embryo and endosperm. In tetraploid *P. simplex*, apomictic seeds show a 2C:5C (4*x*:10*x*) embryo:endosperm genomic ratio, while sexual seeds show a 2C:3C (4*x*:6*x*) ratio (Quarin *et al.*, 1999). Moreover, in apomictic seeds, the endosperm carries an excess of maternal DNA content (4maternal:1paternal) derived from the fertilization of two unreduced polar nuclei (4*x*+4*x*) by a reduced male gamete (2*x*), compared with the sexual ones (2m:1p). The latter is the ratio required for normal endosperm development in many angiosperms and postulated as the EBN (Johnston *et al.*, 1980). All three components of gametophytic apomictic reproduction in *P. simplex* (i.e. apospory, parthenogenesis, and unbalanced endosperm development) always co-segregate and map in a recombinational suppressed area (dubbed the apospory controlling locus, ACL) that presents heterochromatin features (Calderini *et al.*, 2006), deregulation of gene expression (Polegri *et al.*, 2010), and synteny with a conserved genome region of rice and of other grasses (Galla *et al.*, 2019). One of the deregulated genes contained in the ACL shows homology to the *ORIGIN OF REPLICATION COMPLEX* subunit 3 (*ORC3*) (Siena *et al.*, 2016). In yeasts, as well as in higher eukaryotes, the ORC is a six-subunit complex that, together with another multiprotein complex (MCM2–MCM7), and the CDC6 and CDT1 proteins, constitutes the pre-replicative complex (pre-RC), which makes chromatin competent for DNA replication (De Pamphilis, 2003). Within the pre-RC, the ORC is considered as the initiator of DNA replication, being a landing pad for the other components (Bell and Dutta, 2002). Assembled at early G_1_ phase of the cell cycle and acting as a key player for the G_1_/S transition, the pre-RC is considered functionally related to cell proliferation and endoreduplication (Bell and Dutta, 2002). *ORC3* in *P. simplex* exists as three unlinked isogenes of which *PsORC3a*, being specific to apomictic individuals, presents characteristics of a long non-coding RNA (lncRNA). The isogene *PsORC3b*, probably coding for a functional protein, is present in both apomictic and sexual plants, and *PsORC3c*, which codes for a truncated protein, independently segregates from the reproductive mode (Siena *et al.*, 2016). Whereas in sexual plants *PsORC3b* is up-regulated in late phases of endosperm development, it is almost completely silenced in the endosperm of apomictic seeds. This silencing probably depends on a sense/antisense mechanism mediated by *PsORC3a*. Finally, *PsORC3c* is poorly expressed through all the different stages of endosperm development in both sexual and apomictic phenotypes (Siena *et al.*, 2016). Rice and Arabidopsis *orc3* knockedout mutants show normal gametophyte development but arrested embryo and endosperm development at the early stages (Siena *et al.*, 2016). These results indicate that *PsORC3* is one of the crucial genes that control the development of seeds with endosperm harboring an excess of maternal genomes in *P. simplex*. In order to validate this hypothesis, we have tried to down-regulate *PsORC3* in sexual tetraploid genotypes to partially recreate the silencing mechanism present in the natural apomictic *P. simplex* plants, thus obtaining a ‘gain-of-function’ phenotype related to apomictic development in a sexual context. In fact, in sexual genotypes, any deviation from the 2m:1p genome ratio in the endosperm usually leads to seed abortion. Moreover, they lack many other apomixis-linked genes present in the ACL of apomictic genotypes, while they retain the downstream-acting gene machinery necessary to express apomixis fully in crosses. In this context, we have knocked down by RNAi the expression of the *PsORC3* gene in *P. simplex* tetraploid sexual plants either with or without the *PsORC3c* isogene. The resulting transformants have been employed in crosses with diploid pollinators to test their capacity to generate seeds with vital triploid embryos and endosperms with an excess of the DNA maternal content (4m:1p). Our findings confirm that the organization and maternal expression of specific *ORC3* isogenes play a crucial role in controlling the fate of *P. simplex* seeds in which the DNA content of their endosperms deviates from the canonical (2m:1p) ratio.

## Materials and methods

### Plant materials, nomenclature, and growth conditions

Apomictic and sexual tetraploid plants (2*n*=4*x*=40) of *P. simplex* were F_1_ progenies belonging to a mapping population segregating for apomixis (Pupilli *et al.*, 2001), while diploid cytotypes (2*n*=2*x*=20) were collected from a natural population adapted to North-East Argentina (Caceres *et al.*, 1999). Tetraploid sexual plants used as seed donors for transformation experiments were genotyped by PCR for the presence/absence of the *PsORC3c* isogene using specific primers (Supplementary Table S1). Wild-type (WT) plants were grown under routine practices in open greenhouses, whereas transgenic plants were kept in locked cabinets under controlled environmental conditions (80% relative humidity, 8 h daylight, 27–30 °C temperature). Crosses were performed in the cabinet by increasing the relative humidity to 100% and daylight to 12 h (Pupilli *et al.*, 2001). WT and transgenic plants were identified as follows: genotype ID, ploidy level (either 2*n* or 4n), and origin (either WT or RNAi). The suffix c identifies genotypes with (c^+^) or without (c^–^) the *PsORC3c* isogene, or with the bulk of pollen collected from both 2*n* genotypes (c^±^). Regenerated plants derived from transformation with the RNAi vector were named according to the callus from which they originated (callus A, B, or C), followed by a number to identify each single plant. Similarly, plants derived from co-transformation were identified by the callus number (1–78) followed by a suffix A–E.

### Analysis of callus sensitivity to selective agents.

Due to the absence of any established protocol for generating *P. simplex* transgenic plants, we performed preliminary experiments to define a selectable marker on seeds of undefined *PsORC3c* genotype. Embryogenic calli were obtained from mature seeds of open pollinated sexual tetraploid plants of *P. simplex* in isolation as described (Molinari *et al.*, 2003). To test *P. simplex* callus resistance to glufosinate-ammonium (GA), hygromycin (HYG), kanamycin (KN), and paromomycin (PA), these selective agents were added to the growth medium at different concentrations. A growth comparison of 44 one-month-old calli (distributed in four Petri dishes with 11 calli each) in MS2 medium (Molinari *et al.*, 2003) without selective agent (control) or supplemented with GA (0.5 mg l^–1^ or 1.0 mg l^–1^), HYG (50 mg l^–1^ or 75 mg l^–1^), KN (25 mg l^–1^ or 50 mg l^–1^), and PA (25 mg l^–1^ or 50 mg l^–1^) was made. The 396 calli were weighed the first day of the culture and again after 4 weeks on the selective media to measure their growth (Supplementary Fig. S1). The calli were then transferred to shoot proliferation medium MS3 (Molinari *et al.*, 2003) with the corresponding selection agent and, after 4 weeks in culture, the number of calli with shoots was recorded. Preliminary analysis of selective agents (Supplementary Table S2) indicated that HYG 75 mg l^–1^ and GA 1.0 mg l^–1^ were the most effective in retarding callus growth and reducing the percentage of escapes (number of calli that regenerate shoots after an additional 4 weeks in MS3), being 0% for HYG and 9% for GA. Conversely, the callus growth in the presence of KN or PA was not significantly different from that of control calli cultured without any selective agent, even though shoot regeneration was completely inhibited by KN 50 mg l^–1^. Thus, GA or HYG were used as selective agents for transformation experiments.

### Vector construction for nuclear transformation.

A 614 bp fragment of the *PsORC3a* isogene (named *ORC-3frag*), previously used for *in situ* hybridization (ISH) analysis because of the high level of homology with *PsORC3b* and *PsORC3c* (from position 99 to 712 of the *PsORC3a* isogene shown in Siena *et al.*, 2016), was amplified from the cloned sequence of *PsORC3a* by PCR with the forward primer ORfor1 and the reverse primer ORrev1 (Supplementary Table S1) and cloned in the Gateway entry vector pCR™8/GW/TOPO according to the pCR™8/GW/TOPO TA Cloning Kit instructions. The recombinant plasmid pCR™8/GW/TOPO-ORC-3frag was inserted in One Shot TOP10 *Escherichia coli* cells and sequenced to confirm the correct amplification and ligation. Gateway™ LR Clonase™ II enzyme mix was used according to the manufacturer’s instruction to catalyze the *in vitro* recombination between pCR™8/GW/TOPO-ORC-3frag as entry clone and the destination vector pANIC 7B, specifically designed for RNAi-mediated gene suppression in monocots and containing the HYG resistance gene *hph* as selectable marker (Mann *et al.*, 2012). Once the correct assembly of the resulting plasmid pANIC 7B-ORC-3frag (Supplementary Fig. S2) had been confirmed by restriction mapping (not shown), it was used alone, or in combination with plasmid pAHC25 (Christensen and Quail, 1996) which contained the *bar* gene as selectable marker and the *uidA* gene as reporter, for particle bombardment.

### Bombardment and selection of transgenic plants.

To choose the correct shooting parameters, young leaves and calli were bombarded with the plasmid pCK.gfp.S65C, which contained the *gfp* gene under control of the cauliflower mosaic virus (CaMV) 35S promoter (Reichel *et al.*, 1996), using the PDS-1000/He Particle Delivery System (Bio-Rad, Hercules, CA, USA). Applied pressures were 900, 1100, or 1300 psi on leaves and calli placed at 3, 6, or 9 cm from the microprojectile stopping screen. Green fluorescent protein (GFP) was visualized with a Zeiss PALM Microbeam Axio-observer Z1 fluorescence microscope 24 h after bombardment. Images were collected with an AxioCam MRm 60N-C 1″1, ox camera (Zeiss, Oberkochen, Germany) and visualized with Axiovision software (Supplementary Fig. S3). After several assays on both leaves and calli, shooting parameters were fixed at the pressure of 1100 psi and the sample distance from the stopping screen of 9 cm. Seeds used for transformation experiments derived from 53(E) 4*n* WT c^+^ or 39(G) 4*n* WT c^–^ mother plants open pollinated with sexual 4*n* male parents showing PsORC3c^+^ or PsORC3c^–^ genotypes. To generate transgenic plants carrying the RNAi construction, 1-month-old calli from hypocotyl sections were cultured in 9 cm Petri dishes (25–30 calli each dish) onto MS2 medium with 0.4 M sorbitol (for osmotic treatment) for 4 h and bombarded as described (Bellucci *et al.*, 2003). Briefly, 1 µm gold particles were coated with 2.3 μg of pANIC 7B-ORC-3frag DNA; the bombardments were performed as above on samples held at a vacuum of 28 inches of mercury. In total, 390 calli were bombarded. After particle bombardment, the calli were transferred onto MS2 medium (without sorbitol) and incubated in the dark for 1 week at 24 °C. Next, they were selected for 4 weeks onto MS2 medium containing 75 mg l^–1^ HYG, with a 16–8 h photoperiod at 24 °C under 30 μmol m^–1^ s^–2^ light intensity. Subcultures on fresh selective medium were made every 2 weeks. Resistant calli were transferred to MS3 shoot regeneration medium supplemented with 75 mg l^–1^ HYG for 6 weeks and cultured under 150 μmol m^–1^ s^–2^ of light (Supplementary Fig. S4). Lastly, surviving calli were transferred to MS3 without antibiotic and cultivated for other 4 weeks, for a total tissue culture period of 15 weeks. Three calli (named A, B, and C) regenerated shoots rooted in MSO medium (Molinari *et al.*, 2003). For co-transformation experiments, the same method was applied as described above, except that pANIC 7B-ORC-3frag and pAHC25 vectors were mixed in a molar ratio of 2:1 (0.6 μg and 0.18 μg of plasmid DNA, respectively) and 300 calli were bombarded. After particle bombardment, calli were left in MS2 medium with 0.4 M sorbitol for 16 h, then transferred onto MS2 medium and incubated in the dark for 2 d. Afterward, calli were exposed to selection with 1 mg l^–1^ GA onto MS2 medium for 4 weeks and then onto MS3 medium for 4 weeks. Finally, surviving calli were transferred to MS3 without antibiotic and cultivated for a further 2 weeks for a total tissue culture period of 10 weeks and 3 d. Seventy-eight calli regenerated shoots (5–7 shoots for each callus) rooted in MSO medium. Resistant plants were transplanted to pots containing a mixture of soil and peat (1:1) and reared to flower in the greenhouse.

### PCR and Southern analyses

The genomic DNA was extracted with the GenElute™ Plant Genomic DNA Kit (Merck KGaA, Darmstadt, Germany) from ~100 mg of leaf tissue following the manufacturer’s instructions. A 5 µl aliquot of each sample was used as template for PCRs to detect the presence of the transgenes. The amplicons were examined after electrophoresis in a 0.8% agarose gel stained with ethidium bromide (10 mg l^–1^). Similar procedures were adopted for DNA extraction and amplification of the *PsORC3c* isogene. The primer pairs and PCR conditions used for each gene are listed in Supplementary Table S2. Southern blot analysis was carried out as described (Pupilli *et al.*, 2001). In brief, 10 µg of DNA was digested overnight with *Kpn*I, and the resulting fragments were separated by electrophoresis on a 0.8% agarose gel and blotted onto a Hybond-XL nylon membrane (Cytiva UK Limited, Little Chalfont, UK). The 733 bp *gusPlus* and 532 bp HYG fragments used as probes were obtained by amplifying the pANIC 7B-ORC-3frag vector with the primers GUSPlusfw/GUSPlusrev and Hygfw/Hygrev, respectively (Supplementary Table S1). Probe labeling, filter hybridization, washing, and exposure were carried out as reported (Pupilli *et al.*, 2001). For quantitative reverse transcription–PCR (qRT–PCR), total RNA was extracted from florets at 6, 24, 48, 120, and 240 h after pollination (HAP) using the Spectrun™ plant total RNA kit (SIGMA), including a DNase I treatment with the On Column DNase I Digestion set (SIGMA). cDNAs were synthesized from 0.7 µg of total RNA using the SuperScript™ VILO SuperScript kit (Invitrogen; https://www.thermofisher.com/uk/en/home/html).

### qRT–PCR analyses

Primers and Taqman probes for real-time amplification of the *PsORC3b* and *PsORC3c* isogenes and the *CYTIDINE DEAMINASE* housekeeping gene (*PsCDA* GeneBank accession no. AM400871) were those used by Siena *et al.* (2016), under the same concentration and PCR amplification conditions. The suitability of *PsCDA* as a housekeeping gene in *P. simplex* has been tested first in non-saturating RT–PCR experiments (Polegri *et al.*, 2010) and been used subsequently in qRT–PCRs to normalize the expression of specific apomixis-linked genes (Galla *et al.*, 2019) and the different *PsORC3* isogenes in developing sexual and apomictic flowers (Siena *et al.*, 2016). qRT–PCRs were run and analyzed on the 7300 real-time PCR system (Applied Biosystems) according to the manufacturer’s instruction. For each developmental stage and each genotype, two independent RNA isolations from two different inflorescences were carried out. Then, for each RNA sample, two reverse transcription reactions were performed and pooled before running qRT–PCR assays including four technical replicates for each cDNA sample. No-template controls were incorporated in all assays. The relative expression level of *PsORC3* (*b* and *c*) in each genotype at each developmental stage was calculated with the ΔCt method (Pfaffl, 2001). For each experiment, the normalized quantity of the target gene in the sample 53(E) 4*n* WT c^+^ was used as a calibrator (6HAP in Fig. 3A, 120 HAP in Fig. 3B, and 4*n*×2*n* in Fig. 3C), and arbitrarily set to 1. Then the relative expression levels of the target gene in all other samples were expressed in relation to this control. Target expression values of all samples were analyzed for normality with Shapiro–Wilk W-test. Then, averages values were compared by ANOVA. The Duncan test was run for estimating significant difference between samples at *P*<0.05. Statistical analyses were carried out using the Infostat Software Package (Di Rienzo *et al.*, 2017).

**Fig. 1.**
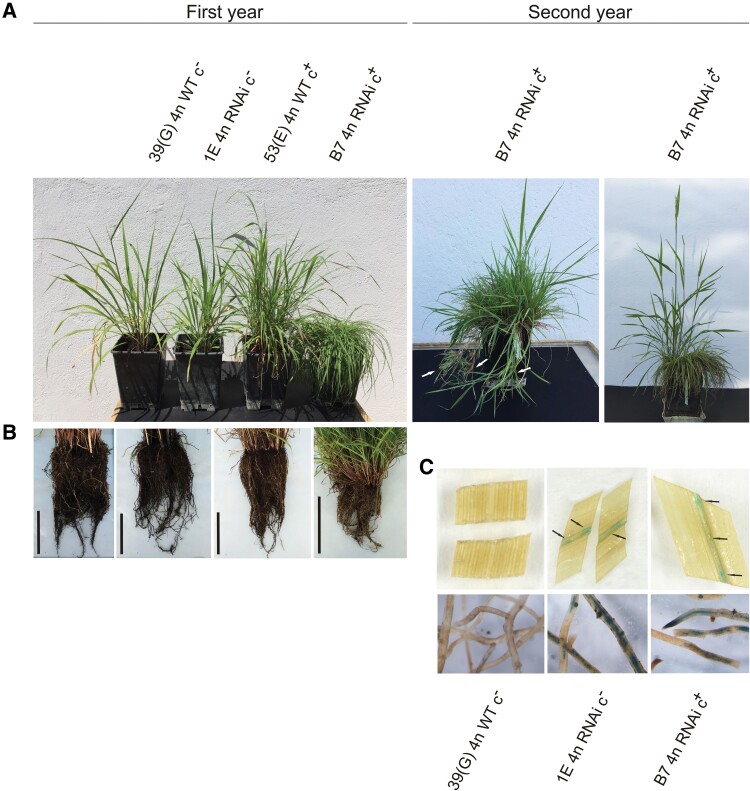
Phenotypic analysis of the *P. simplex* transformed plants. (A) Representative photographs of the aerial part of wild type [39(G) 4*n* WT c^–^ and 53(E) 4*n* WT c^+^] and RNAi transgenic (1E 4*n* RNAi c^–^ and B7 4*n* RNAi c^+^) plants in the first and second year of growth. In the second year, B7 4*n* RNAi c^+^ developed a transient phenotype with stoloniferous structures (white arrows), which disappeared later when this plant developed reproductive branches (second year, right picture). (B) Root apparatus of WT and RNAi genotypes; black bar length is 10 cm. (C) GUS colorimetric assay on sections of plant leaves and roots. Black arrows indicate the blue color of the transformed plant vascular tissue.

**Fig. 2.**
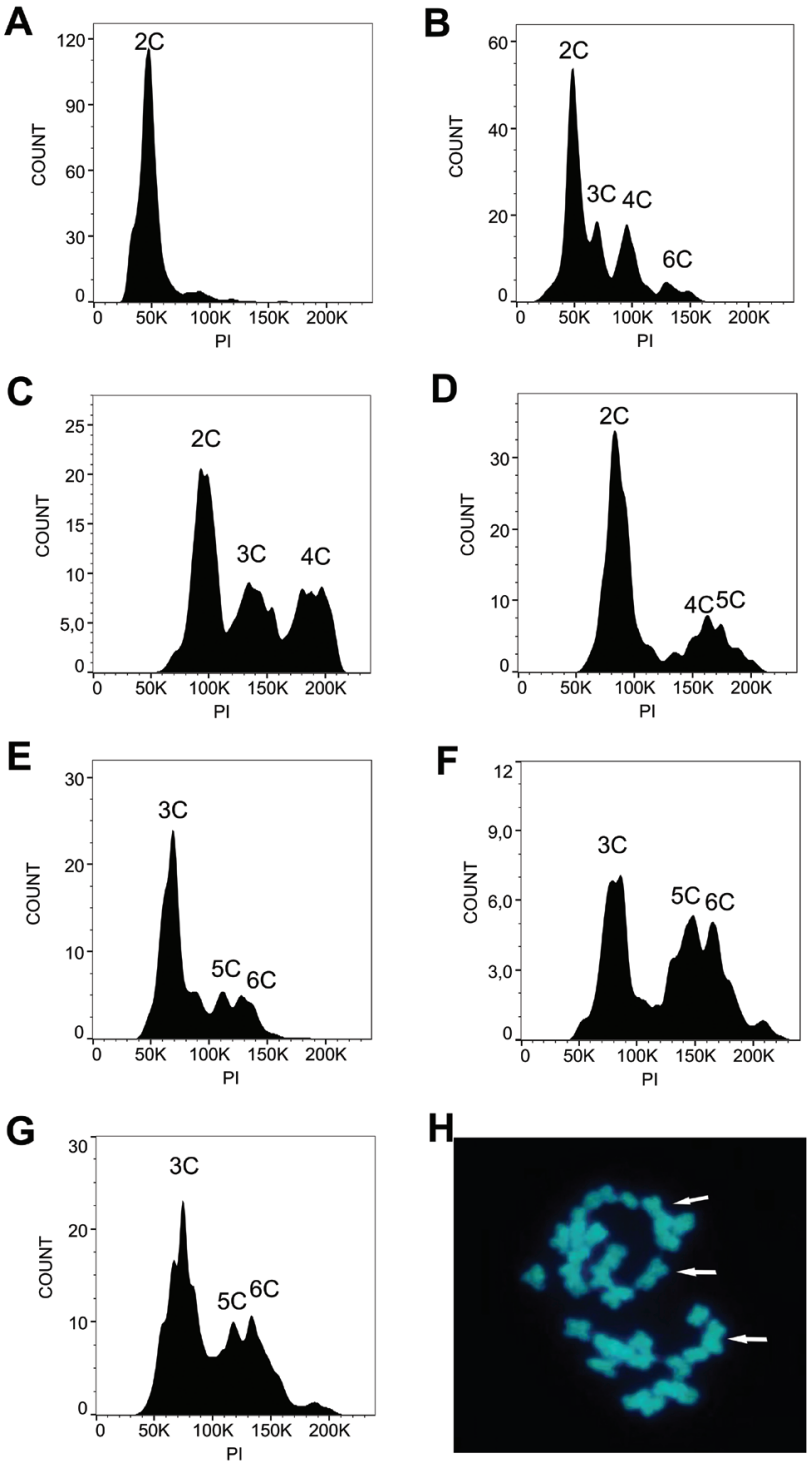
Flow cytometric analysis of mature seeds and chromosome counts in *P. simplex* hybrids. (A) Histogram of leaf tissue from a control diploid (2*n*=2*x*) individual. (B) Histogram of mature seed of a diploid cytotype. The 2C (2*x*) peak corresponds to the DNA content of nuclei in the embryo and the smaller 3C peak to nuclei in the endosperm. The 4C and 6C peaks correspond to nuclei from the embryo and endosperm that have undergone endoreduplication. (C) Histogram of a sexual tetraploid (2*n*=4*x*) seed showing a 2C peak corresponding to the embryo (4*x*) and a smaller peak related to the endosperm 3C (6*x*). The 4C peak accounts for the endoreduplication of some nuclei of the embryo. (D) Histogram of a WT pseudogamous apomictic tetraploid seed showing a 2C peak corresponding to a parthenogenetic embryo (4*x*) and a 5C peak corresponding to a maternal excess endosperm; the 4C peak is related to embryo endopolyploidization. (E) Histogram of a seed derived from the 1E 4*n* RNAi c^–^×Bulk 2*n* WT c^±^ interploidy cross (Table 2) showing a 3C:5C peak pattern. This embryo:endosperm genome ratio is derived from the double fertilization of a reduced (*n*=2*x*) egg cell and both polar nuclei (*n*=2*x*+*n*=2*x*) by reduced (*n*) male gametes to form a triploid embryo (2*n*+*n*) and a pentaploid (2*n*+2*n*+*n*) maternal excess endosperm. (F) Histogram of a seed derived from the B7 4*n* RNAi c^+^×Bulk 2*n* WT c^±^ interploidy cross (Table 2) showing again a 3C:5C peak pattern. (G) Histogram of seed derived from the 27(C.1) 4*n* WT c^–^×D9 2*n* WT c^+^ control interploidy cross (Table 2) showing a 3C:5C pattern similar to that described in (E). A 6C peak in (E–G) can be attributed to endoreduplication of the embryo cells. (H) Root tip metaphase chromosome spread of a seed from the control cross described in (G). Arrows indicate three metacentric homologous chromosomes. PI, propidium iodide sensor.

**Fig. 3.**
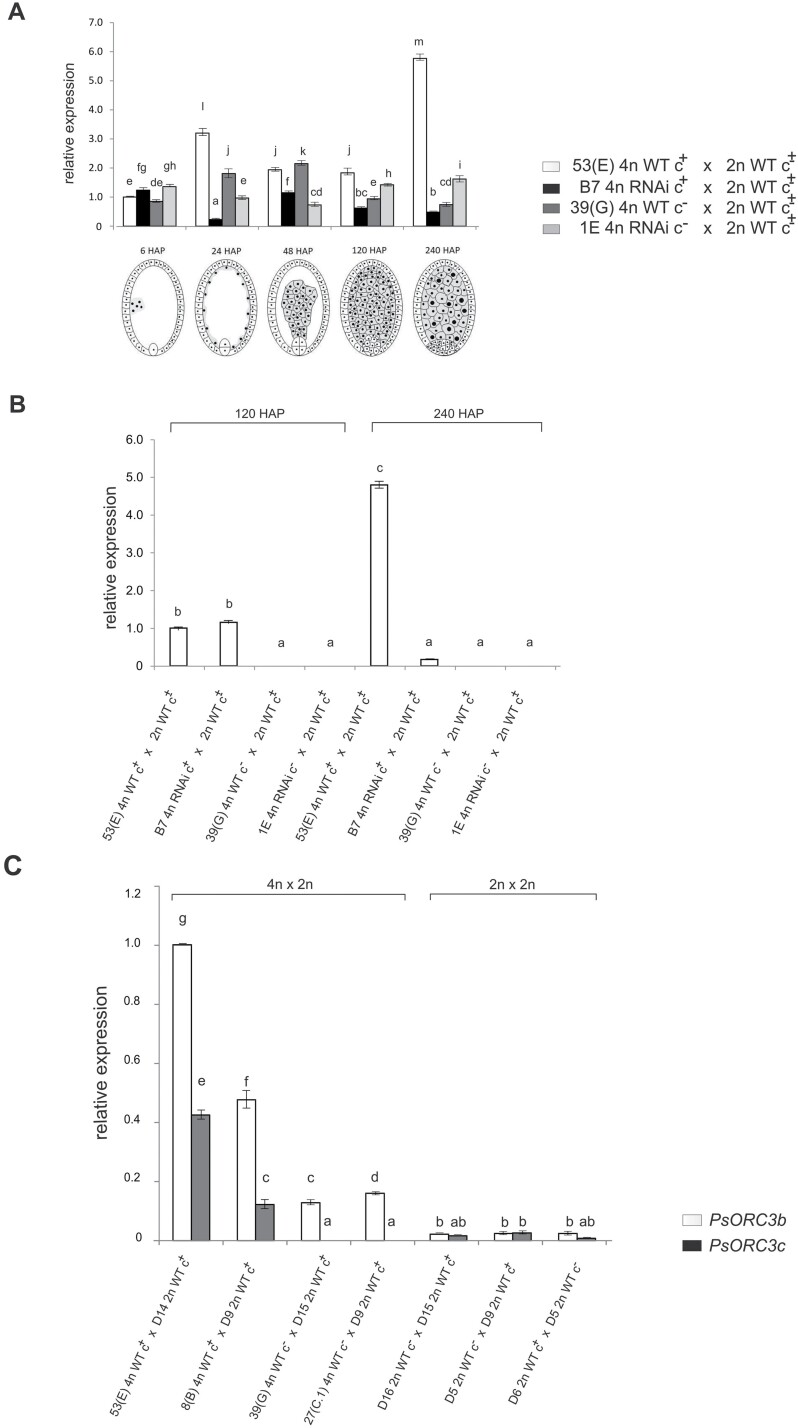
qRT–PCR transcriptional profiles of *PsORC3b* and *PsORC3c* isogenes in developing endosperms of *P. simplex*. (A) Relative expression profile of the *PsORC3b* isogene in interploidy crosses of WT and RNAi-inactivated tetraploid mother plants pollinated with diploid genotypes. The numbers followed by HAP indicate the hours after pollination of sample collection and mark the endosperm developmental stages schematized below the graph corresponding to: 6 HAP (first mitotic divisions), 24 HAP (syncytium with nuclei localized at periphery of the embryo sac), 48 HAP (late cellularization), 120 HAP (late proliferative stage), and 240 HAP (onset of endoreduplication). (B) Relative expression profile of *PsORC3c* in the same material as in the last two stages of (A). (C) Relative expression profile of *PsORC3b* and *PsORC3c* isogenes in inter- and homoploid crosses in which the mother plant differs for the presence of the *PsORC3c* isogene. The same letters on the top of the histograms indicate non-significant differences for the expression for *P*≤0.005, *c*^+^ or *c*^–^ indicate presence or absence of the *PsORC3c* isogene in the parent lines, and *c*^±^ indicates bulked pollen collected from mixed 2*n* genotypes. Bars on the top of histograms indicate the SEs. The relative expression level refers to the expression value of the *PsORC3b* isogene of the sample 53(E) 4*n* WT c^+^ in (A) (6 HAP) and (C), and of the *PsORC3c* isogene of the same sample in (B) arbitrarily set to 1.

### Histology

For early seed histological analysis, inflorescences were collected at pre-meiosis, anthesis, and then at 3, 6, 12, 24, 48, 120, and 240 HAP, and fixed in absolute ethanol–acetic acid 3:1 (v/v) for at least 24 h. Fixed samples were dehydrated in an ethanol series, cleared in xylene, and embedded in Paraplast (Sigma). Each flower was longitudinally sectioned with a rotary microtome in slices of 12 µm. Approximately half of the sections were stained with safranin and fast green mixture (Caceres and Mazzuccato, 1995) for megasporogenesis, megagametogenesis, and endosperm development analyses.

### Flow cytometric analysis

Spikelets were removed from the rachis 30 d after pollination, and filled seeds were manually selected. Caryopses were separated from the glumes, and their nuclei were extracted by chopping the grains with a razor blade into 0.1 ml of the extraction buffer ‘Otto I’ [100 mM citric acid, 0.5% (v/v), Tween 20, pH 2–3] (Otto, 1992). After 5 min, suspensions of nuclei were mixed with 0.24 ml of the staining buffer ‘Otto II’ (400 mM Na_2_PO_4_·12H_2_O, pH 8) and filtered through a 30 mm nylon filter to remove cell fragments and large debris. Then, 7 µl of propidium iodide (PI) (Sigma, P4170) (1 g l^–1^) and 50 g l^–1^ RNase I (Sigma, St Louis, MO, USA) were added to each sample. After 10 min incubation at room temperature, fluorescence intensity of PI-stained nuclei was measured by a BD FACSAria II cytometer (BD Biosciences, San Jose, CA, USA) with excitation and emission wavelengths of 538 nm and 617 nm, respectively (Otto, 1992). Data were analyzed by FlowJo v.10.0.7 software (BD Biosciences). A minimum of 1000 nuclei (within the scoring window) were analyzed in each sample. Developmental pathways of seed formation were estimated by applying the FCSS method as described (Matzk *et al.*, 2001; Doležel *et al.*, 2007). Seeds were individually analyzed, and ploidy levels of the embryo and endosperm were estimated by comparing different histogram peaks. Diploid (2*n*=2*x*=20) leaf tissue from a *P. simplex* individual, together with seeds derived from previously classified sexual [27 (C.1) 4*n* WT c^–^] and apomictic (31A 4*n* WT c^+^) plants were used as internal controls.

### Chromosome counts

At least 10 mature seeds for each plant were soaked in 0.01% SDS for 10 min at room temperature, and then germinated on moist filter paper in Petri dishes. Root apices (5 mm long) were treated with 0.01% colchicine (Sigma) at room temperature for 2 h and fixed in ethanol–acetic acid 3:1 (v/v) for at least 24 h. Later, fixed root tips were treated with a solution of 10% pectinase (Sigma), 2% macerozyme (Serva), and 8% cellulase (Calbiochem) in citrate buffer pH 4.6 for 50 min at 37 °C and squashed under a coverslip in a drop of 60% acetic acid. The coverslips were removed after freezing at –80 °C. The air-dried preparations were stained with 0.2 µg ml^–1^ DAPI in McIlvaine buffer pH 7.0 and mounted in AF1 antifade solution (Citifluor). Observation was carried out using a Leica DMRB fluorescence microscope and images were captured by an ILCE-7 camera (SONY).

### Feulgen analysis

Three developing seeds at 120 HAP were collected from each sexual and apomictic genotype and fixed in ethanol–acetic acid 3:1 for at least 24 h at 4 °C. Fixed developing seeds were treated with a mixture of 10% pectinase (Sigma), 2% macerozyme (Serva), and 10% cellulase (Calbiochem) in citrate buffer pH 4.6 for 90 min at 37 °C and squashed in gelatinized slides under a coverslip in a drop of 45% acetic acid. Squashes were then hydrolyzed in 1 N HCl at 60 °C for 15 min, Feulgen-stained, and washed for 10 min in SO^2^-water (three changes) prior to dehydration in ethanol:xylene series and mounting in DPX (BDH Chemicals). Feulgen-stained DNAs in individual nuclei wwew measured in images captured by a charge-coupled-device camera on a Leica DMRB microscope, using a Leica Q500MC image analyzer.

## Results

### Generation of transgenic plants carrying the ORC3 RNAi cassette

To target *PsORC3b* by RNAi, a protocol had been developed for stable genetic transformation of tetraploid sexual individuals of *P. simplex* through particle bombardment, and transgenic plants of this species were obtained for the first time. The role of *PsORC3b* in endosperm development was evaluated in the two genetic constitutions characterizing the *PsORC3* gene in the sexual background (*PsORC3b* alone or in combination with *PsORC3c*). Thus, sexual genotypes with [53(E) 4*n* WT c^+^] and without [39(G) 4*n* WT c^–^] the *PsORCc* isogene (Supplementary Fig. S5A) were used as seed donors for transformation experiments. HYG and GA were chosen as the most effective selecting agents based upon preliminary experiments on their ability to retard callus growth (see the Materials and methods and Supplementary Fig. S1 and Supplementary Table S2).

The RNAi vector pANIC 7B-ORC-3frag designed to down-regulate *PsORC3* expression (Supplementary Fig. S2A) was employed alone or in combination with plasmid pAHC25 (Supplementary Fig. S2D). In a first experimental set, 390 calli derived from 53(E) 4*n* WT c^+^ seeds were bombarded with pANIC 7B-ORC-3frag vector and selected with HYG 75 mg l^–1^. After being selected and transferred to MS3 medium, only three calli (named A, B, and C) succeeded in regenerating plants. A total of 28 plants (six from callus A, 17 from callus B, and five from callus C) were grown to maturity and investigated for the presence of the *hph* and *gusPlus* genes, as well as the two ORC-3frag fragments placed in opposite orientation (Supplementary Fig. S2A). PCR analyses showed that all plants derived from the calli A and B amplified the fragment corresponding to the selectable marker *hph* coding for HYG resistance (Table 1). Nevertheless, none of the plants from callus A showed the ORC-3frag amplicons and only 14 plants from callus B (B3, B4, B7–B14, and B30–B33) amplified the ORC-3frag-specific fragments in the correct orientation (Supplementary Fig. S2B, C; Table 1). The five plants derived from callus C failed to amplify any of the transgenes and thus they were considered as escapes (Supplementary Fig. S2B, C; Table 1). According to these results, the transformation efficiency, calculated as the number of independent transformed calli (2) divided by the total number of bombarded calli (390), was 0.5% or 0.25% only if the genomic incorporation of the RNAi cassette was taken into account.

**Table 1.** PCR analysis of regenerated plants

Hygromycin (HYG)						
Callus	Plant	Gene/fragment				
		*hph* ^a^	ORC-3a (antisense orientation) ^a^	ORC-3a (sense orientation) ^a^	*GUSPlus* ^a^	
A	A1–A6	**+**	**–**	**–**	**–**	
B	B1, B5, B6	**+**	**–**	**–**	**–**	
	B3, B4, B7–B14, B30–B33	**+**	**+**	**+**	**+**	
C	C1–C5	**–**	**–**	**–**	**–**	
**Glufosinate-ammonium (GA)**						
**Callus** ^**b**^	**Plant**	**Gene/fragment**				
		***hph***	**ORC-3a (antisense orientation)**	**ORC-3a (sense orientation)**	***GUS*** ^**a**^	***bar*** ^**a**^
1	1A, 1C	**–**	**+**	**+**	**–**	**–**
	1B, 1D, 1E	**+**	**+**	**+**	**+**	**+**
2–78		**–**	**–**	**–**	**–**	**–**

The hygromycin panel includes plants derived from transformation with pANIC 7B-ORC-3frag selected with hygromycin 75 mg l^–1^. The glufosinate-ammonium panel includes plants derived from co-transformation with plasmids pANIC 7B-ORC-3a and pAHC25 selected with glufosinate-ammonium 1 mg l^–1^.

^a^ Primer pairs used to amplify each gene/fragment are reported in Supplementary Table S1.

^b^ For each of the 78 calli, the DNA was extracted from five regenerated plants, bulked, and PCR analyzed.

For each gene/fragment considered, the presence (+) or absence (–) of the expected amplicon is indicated.

To obtain transgenic plants in the *PsORC3c*^*–*^ background, 300 calli derived from 39(G) 4*n* WT c^–^ seeds were bombarded with a mixture of the pANIC 7B-ORC-3frag and pAHC25 vectors (co-transformation strategy), and then selected with GA 1 mg l^–1^. In total, 78 resistant calli (numbered from 1 to 78) and 5–7 plants for each callus were obtained. The PCR screening for the presence of both the *hph* and *ORC-3frag* DNA sequences, carried by the pANIC 7B-ORC-3frag vector, and of both the *bar* and *GUS* (β-glucuronidase) fragments, present in the pAHC25 plasmid, showed that only three plants derived from callus 1 were positive for all the fragments tested (transformation efficiency 0.3%; Supplementary Fig. S2E–G; Table 1). This meant that plants 1B, 1D, and 1E had incorporated both transformation vectors. Another two plants (1A and 1C) amplified only the *ORC-3frag* fragments present in the RNAi construct (Table 1). The occurrence of transformed plants showing an incomplete nuclear integration of transgenic cassettes was not surprising because the biolistic method in monocots could generate plants containing complex arrays of rearranged plasmids (Register *et al.*, 1994; Liu *et al.*, 2019). The plants regenerated from the other 77 calli without amplifying any of the transgene fragments were considered as escapes (Table 1).

To estimate the number of transgene insertions in transformed plants, blots of genomic DNA digested with *Kpn*I (that cuts the pANIC 7B-ORC-3frag vector only once, upstream of the two ORC-3frag copies Supplementary Fig. S2A), were hybridized with *gusPlus* and *hph* probes. The hybridizing pattern of the *gusPlus* probe showed three bands of ~17.0, 15.6, and 10.0 kb in plants 1B, 1D, and 1E (Supplementary Fig. S6A), indicating that the pANIC 7B-ORC-3frag had been inserted in three different sites of the genome. The hybridizing pattern of plants 1B and 1D with the *hph* probe showed the same three bands (Supplementary Fig. S6B), confirming the presence of three integrated copies of pANIC 7B-ORC-3frag in these individuals. Likewise, an identical hybridization pattern consisting of two bands was shown by the transgenic plants derived from callus B (i.e. B3, B4, B7–B14, and B30–B33) when hybridized with the *gusPlus* probe (Supplementary Fig. S6A). Two bands were also shown by B3, B7, B8, and B9 plants when hybridized with the *hph* probe (Supplementary Fig. S6C), indicating that B plants had incorporated two copies of the pANIC 7B-ORC-3frag vector. Southern analysis also revealed that transformants carrying rearrangements of the same vector (plants A1 and B1, Supplementary Fig. S6A, C) probably originated from independent regeneration events in callus A and B, respectively. However, being PCR-negative for ORC-3frag amplicons, these two plants were not further investigated.

In conclusion, two transgenic genotypes derived from two independent transformation events were obtained: one (generating the clonal plants 1B, 1D, and 1E) derived from co-transformation of explants from seeds obtained from a mother plant holding a PsORC3c^–^ genotype, and the other (clonal plants B3, B4, B7–B14, and B30–B33), came from transformation of seed-derived explants from a PsORC3c^+^ mother plant.

### Phenotype characterization of the ORC3 RNAi lines

Based on the results described above, two transgenic plants from callus B (B7 and B31) and two from callus 1 (1B and 1E) were analyzed by PCR to determine the presence/absence of the *PsORCc* isogene. The latter was present in both transgenic plants from callus B but absent in the two plants derived from callus 1. After genotyping, transgenic plants, renamed B7 4*n* RNAi c^+^, B31 4*n* RNAi c^+^, 1B 4*n* RNAi c^–^, and 1E 4*n* RNAi c^–^ (Supplementary Fig. S5B), were used for phenotype characterization. Since no phenotypic difference had been detected among plants derived from the same transformation event, only one representative for each event was reported in Fig. 1. The transgenic plants 1E 4*n* RNAi c^–^ and 1B 4*n* RNAi c^–^, very similar to their related WT [39(G) 4*n* WT c^–^] for both the aerial part and root system (Fig. 1A, B), developed reproductive structures within the first year of acclimation in the growth chamber. In contrast, B7 4*n* RNAi c^+^ and B31 4*n* RNAi c^+^ showed a prostrate habitus and a strongly reduced root system compared with their related WT [53(E) 4*n* WT c^+^; Fig. 1A, B]. The prostrate habitus lasted throughout the first year, when these plants were not able to differentiate reproductive branches. However, during the following year, they first generated some transient stoloniferous structures and later erect reproductive branches (Fig. 1A, second year; B7 4n RNAi c^+^), reaching full bloom during the third year. The consistent differences detected between plants derived from the two transformation events could probably be ascribed to the longer tissue culture phase of the transformation experiments (15 weeks) compared with the co-transformation experiments (11 weeks). Based on the vectors used for their transformation, these plants should have expressed the *gus* and/or the *gusPlus* gene and, as expected, the GUS colorimetric assay showed prevalent expression of this marker on leaf vascular tissues and young root apexes (Fig. 1C).

### Fertility of interploidy crosses in WT and transgenic plants

A series of homo- and interploidy crosses were performed to determine the ability of sexual ORC3 RNAi plants to develop seeds with maternal genome excess endosperm, which could possibly be correlated with *PsORC3* inactivation (Table 2). The transgenic plants derived from callus B (B7 4*n* RNAi c^+^ and B31 4*n* RNAi c^+^) and callus 1 (1B 4*n* RNAi c^–^ and 1E 4*n* RNAi c^–^), along with their tetraploid seed donor plants 53(E) 4*n* WT c^+^ and 39(G) 4*n* WT c^–^, respectively, were used as maternal parents in crosses with diploid plants of mixed PsORC3c^+^ and c^–^ genotypes (Table 2). As expected, and in agreement with the EBN model (Johnston *et al.*, 1980), the control plant 53(E) 4*n* WT c^+^ did not form seeds after these (interploidy) crosses. In stark contrast, the four *ORC3* RNAi plants did set seeds, albeit in a low number, suggesting that transformation events might have caused the relaxation of the mechanisms controlling triploid block in *P. simplex.* Yet, a relaxation of the triploid block should also be used to explain the presence of a consistent number of seeds in the interploidy crosses when the other donor plant, the control 39(G) 4*n* WT c^–^, was employed. These results raised the question of whether the presence of the *PsORC3c* isogene in the mother parent might be associated with the arrest of seed development in interploidy crosses. In order to examine this hypothesis, interploidy crosses between flowers of sexual tetraploid genotypes, either with or without *PsORC3c*, and pollen from single diploid plants harboring the *PsORC3c* isogene were performed (Table 2, ‘Additional control crosses’). Interestingly, seeds were obtained only from those crosses in which mother plants carried the PsORC3c^–^ genetic constitution, while no seed was produced when they were PsORC3c^+^.

**Table 2.** Cross design and seed set in hybridization of *P. simplex*

Parent composition^a^		No. of crosses	Obtained seed	Genome composition^b^	
♀	♂			Expected	Estimated, no. of seeds analyzed by flow cytometry
Transformation					
53(E) 4*n* WT c^+c^	Bulk 2*n* WT c^±^	145	0	3C:5C (4m:1p) ^d^	–
B7 4*n* RNAi c^+^	Bulk 2*n* WT c^±^	230	4	3C:5C (4m:1p)	3C:5C (4m:1p), 3; 3C:4C:5C (4m:1p), 1
B31 4*n* RNAi c^+^	Bulk 2*n* WT c^±^	187	2	3C:5C (4m:1p)	3C:5C (4m:1p), 2
Co-transformation					
39(G) 4*n* WT c^– c^	Bulk 2*n* WT c^±^	298	25	^d^3C:5C (4m:1p)	3C:5C (2m:1p), 5
1E 4*n* RNAi c^–^	Bulk 2*n* WT c^±^	238	4	3C:5C (4m:1p)	3C:5C (4m:1p), 4
1B 4*n* RNAi c^–^	Bulk 2*n* WT c^±^	196	5	3C:5C (4m:1p)	3C:5C (4m:1p), 3; 2C:3C (2m:1p), 2
Additional control crosses					
53(E) 4*n* WT c^+^	D14 2*n* WT c^+^	83	0	^d^3C:5C (4m:1p)	–
08(B) 4*n* WT c^+^	D9 2*n* WT c^+^	157	0	^d^3C:5C (4m:1p)	–
32(I) 4*n* WT c^+^	D15 2*n* WT c^+^	209	0	^d^3C:5C (4m:1p)	–
39(G) 4*n* WT c^–^	D15 2*n* WT c^+^	130	15	^d^3C:5C (4m:1p)	3C:5C (4m:1p), 5
27(C.1) 4*n* WT c^–^	D9 2*n* WT c^+^	276	28	^d^3C:5C (4m:1p)	3C:5C (4m:1p), 5
28(Y) 4*n* WT c^–^	D14 2*n* WT c^+^	189	6	^d^3C:5C (4m:1p)	3C:5C (4m:1p), 6
D5 2*n* WT c^–^	D9 2*n* WT c^+^	145	12	2C:3C (2m:1p)	2C:3C (2m:1p), 5
D16 2*n* WT c^–^	D15 2*n* WT c^+^	89	16	2C:3C (2m:1p)	2C:3C (2m:1p), 5
D6 2*n* WT c^+^	D5 2*n* WT c^–^	79	10	2C:3C (2m:1p)	2C:3C (2m:1p), 4

^a^ Genotypes with (c^+^) or without (c^–^) *PsORC3c* isoform; bulk of pollen (c^±^) collected from both genotypes.

^b^ Embryo:endosperm genome ratio (endosperm genome composition, maternal:paternal).

^c^ Control cross.

^d^ Crosses that are not expected to set seeds.

### Validation of the triploid nature of embryo and maternal excess genome contribution of the endosperm of the seeds derived from interploidy crosses

Seeds derived from interploidy crosses were analyzed by flow cytometry, to verify the triploid nature of the embryo and estimate the maternal:paternal genome contribution in the endosperm. Furthermore, somatic chromosome counts were carried out on a subsample of these seeds to corroborate the flow cytometry results (Fig. 2). Leaf material from diploid genotypes as well as mature seeds collected from both apomictic and sexual open-pollinated plants were included in the analysis as controls. Seeds derived from sexual (diploid and tetraploid, Fig. 2B and C respectively) and apomictic (Fig. 2D) controls showed the expected histograms, with 2C:3C and 2C:5C peaks, respectively. Endoreduplication occurred in both embryo and endosperm tissues, producing additional peaks with values that resulted in multiples of the basic (2C and 3C) DNA content (Fig. 2B–G). In some cases, due to adjustment of the flow cytometer and variations in the fluorescence of each sample, comparable peaks failed to exactly coincide positionally in the abscissa, but the ratio of the embryo and endosperm DNA content in cells always remained constant for each type of seed. The flow cytometric analysis of all four seeds derived from the mother plant 1E 4*n* RNAi c^–^ showed an embryo:endosperm DNA content ratio of 3C:5C (Table 2; Fig. 2E). This histogram could only be shown by seeds with a triploid embryo (2*n*=3*x*), originated from the fertilization of a reduced egg cell of the tetraploid RNAi line (*n*=2*x*) with a reduced male gamete of the diploid pollinator (*n*=*x*), and a pentaploid endosperm (5C=5*x*) derived by the fusion of the two reduced polar nuclei of the central cell (*n*=2*x*+*n*=2*x*) with the second reduced male gamete (*n*=*x*). Similar results were obtained with three out of four seeds derived from mother plant B7 4*n* RNAi c^+^ (Table 2; Fig. 2F), three out of five seeds of 1B 4*n* RNAi c^–^, as well as the two seeds of B31 4*n* RNAi c^+^ (Table 2). Seeds from plant 1B 4*n* RNAi c^–^ showing a 2C:3C peak histogram probably derived from self-fertilization events (Table 2). In the B7 4*n* RNAi c^+^ mother plant, a seed showing a histogram with an extra 4C peak was also detected (Table 2). A subset of seeds derived from crosses between WT tetraploid plants, without the isogene *PsORC3c* (Table 2), and diploid pollen donors consistently showed histograms with 3C:5C peaks, indicative of seeds with maternal genome excess in the endosperm (Fig. 2G). Moreover, homoploid (2*n*×2*n*) control crosses showed in all cases the expected histograms with 2C:3C peaks (not shown). On the other hand, chromosome counts on root tips of a subsample of progenies obtained from the mother plants 27(C.1) 4*n* WT c^–^ and 39(G) 4*n* WT c^–^ crossed with diploid genotypes harboring *PsORC3c* (Table 2) showed the expected number of 30 chromosomes, with three copies of a longer metacentric chromosome, each presumably belonging to a single chromosome complement (*n*=1, *x*=10), which was easily recognizable (Fig. 2H, arrows). In summary, seeds with a triploid embryo and maternal excess endosperm were collected from all manipulated *ORC3* plants as well as WTs lacking the *PsORC3c* isogene when they were pollinated by diploid individuals.

### The generation of triploid seeds with maternal excess endosperm is functionally related to down-regulation of *PsORC3b* and *PsORC3c*

If the silencing of *PsORC3* by RNAi could account for seed setting when transgenic plants were crossed with diploid pollen donors, the arrest of seed development in control interploidy crosses would conversely be due to the expression of the *PsORC3c* isogene in the maternal parent. In order to test these hypotheses, isogene-specific real-time PCR analyses for *PsORC3b* and *PsORC3c* were carried out in developing seeds derived from interploidy crosses involving WT and transgenic RNAi plants, either with or without the *PsORC3c* isogene, as mother plants and diploid genotypes as pollinators. Five developmental stages were taken into account: early endosperm mitotic divisions (6 HAP), end of the syncytium formation (24 HAP), start-to-end of cellularization (48 HAP), completion of mitotic/proliferative phase (120 HAP), and early phases of endoreduplication (240 HAP) (Fig. 3A). Since the cross combinations reported in Fig. 3A and B shared the same bulk of pollen from diploid plants carrying both PsORC3c^+^ and PsORC3c^–^ genotypes (c^±^), the developing endosperms were here identified by the name of their mother plants. In the control plant 53(E) 4*n* WT c^+^, *PsORC3b* reached the minimum transcript levels at stage 6 HAP, and then peaked at stage 24 HAP to hit their maximum at 240 HAP. Interestingly, the *PsORC3b* transcript levels in developing seeds of the RNAi plant (B7 4*n* RNAi c^+^) were significantly lower than the related control from stage 24 HAP onwards. This suggested that RNAi was effective in down-regulating *PsORC3b* expression in this transformation event if its transcripts were raised to the basal level at stage 6 HAP. Similarly, *PsORC3b* was down-regulated in leaves of RNAi plants compared with their related untrasformed control (Supplementary Fig. S7). In developing seeds collected from the WT lacking the isogene *PsORC3c* [39(G) 4*n* WT c^–^], the *PsORC3b* expression was significantly reduced at stages 24, 120, and 240 HAP, with a single small up-regulation at stage 48 HAP, with respect to those collected from the control plant with *PsORC3c* [53(E) 4*n* WT c^+^]. It is likely that the presence of *PsORC3c* boosted the *PsORC3b* expression pattern. Interestingly, in seeds from 1E 4*n* RNAi c^–^, *PsORC3b* was significantly down-regulated with respect to its recipient genotype [39(G) 4*n* WT c^–^] at stages 24 and 48 HAP, whereas it was up-regulated at subsequent stages, when the *PsORC3b* transcript levels of the recipient genotype reached the minimum. It is noteworthy that in both RNAi lines, the expression of *PsORC3b* in the seeds was significantly reduced with respect to their controls at stages 24 and 48 HAP, corresponding to the end of syncytium formation, and start-to-end of cellularization (48 HAP), respectively. Conversely, at the later stages, the RNAi cassette seemed to have made an impact on the expression of *PsORC3b* depending on whether the seed parent harbored the *PsORC3c* isogene. So, we decided to investigate the transcript levels of *PsORC3c* in the same samples stated above at 120 and 240 HAP (Fig. 3B). *PsORC3c* was expressed only in the seeds whose mother plants carried the isogene. Notably, its expression significantly increased from 120 to 240 HAP only in the control line, but not in B7 4*n* RNAi c^+^, which, on the contrary, showed a drastic reduction in expression at this phase of seed development. Thus, RNAi transformation strongly reduced the expression of both *PsORC3b* and *c* isogenes at early phases of endoreduplication (240 HAP) in immature seeds collected from PsORC3c^+^ mother plants. Moreover, *PsORC3c* expression was not detected even when the male parent could have contributed this isogene (Fig. 3B). The latter result could be attributed either to a poor transmission of *PsORC3c* from the male parent or to its parent-of-origin expression. To discriminate between these two possibilities, tetraploid mother plants (either c^+^ or c^–^) were fertilized with pollen collected only from diploid PsORC3c^+^ genotypes, and qRT–PCR analysis was performed on the developing seeds (Fig. 3C). In this way, assuming a high heterozygosity of the diploid male parent due to strict outcrossing (Caceres *et al.*, 1999), *PsORC3c* was expected to segregate 1:0 (presence:absence) in the male gametes and thus to be transmitted at a maximum in 50% of progenies in crosses with a mother plant null for this isogene. Since the highest expression of both *PsORC3b* and *c* was detected at the latest stage of seed development, only this stage was taken into consideration for qRT–PCR. Homoploidy crosses at the diploid level were also included as controls (Fig. 3C). Again, high expression of *PsORC3c* was detected only in immature seeds resulting from interploidy crosses in which the mother plants were c^+^ genotypes [53(E) 4*n* WT c^+^ and 8(B) 4*n* WT c^+^], whereas no expression was detected in those hybrid seeds whose mother plants were null for the same isogene [39(G) 4*n* WT c^–^ and 27(C.1) 4*n* WT c^–^]. Strikingly, *PsORC3c* expression was detected, albeit at low levels, in all three homoploid crosses performed, regardless of whether the mother plants were null for *PsORC3c* (D16 and D5) or not (D6). This suggested that in interploidy crosses *PsORC3c* was expressed when inherited from the maternal parent but was silenced if inherited from the paternal parent. Furthermore, *PsORC3b* mRNA reached the highest levels in those samples expressing *PsORC3c*, to drop in seeds from interploidy crosses whose mother plants were null for *PsORC3c*. Conversely, in homodiploidy crosses, *PsORC3c* as well as *PsORC3b* were always moderately expressed, regardless of the presence of *PsORC3c* in the mother plant (Fig. 3C). So as to verify the transmissibility of *PsORC3c* through male gametes, plants were raised from seeds generated by both interploidy and homoploidy crosses between *PsORC3c*^*–*^ (tetraploid or diploid) seed parents and *PsORC3c*^*+*^ diploid male parents. Approximately half of the progenies harbored *PsORC3c* in both 4*n*×2*n* and 2*n*×2*n* control crosses, thus confirming its full transmissibility through male gametes (Supplementary Fig. S8). Overall, the results indicated that *PsORC3c* had undergone a maternal-specific expression, at least in interploidy crosses. In conclusion, the production of triploid seeds with maternal excess endosperm in sexual *P. simplex* became possible only when *PsORC3c* was absent or silenced and *PsORC3b* was down-regulated. In turn, the present data suggested that the transcript levels of *PsORC3b* and *PsORC3c* appeared to be a key determinant for further development of seeds with a maternal to paternal genome contribution deviating from the EBN.

### Maternal excess endosperms from PsORC3 RNAi plants succeeded in overcoming the arrest on development of unbalanced endosperm at the proliferative phase prior to endoreduplication

**Fig. 4.**
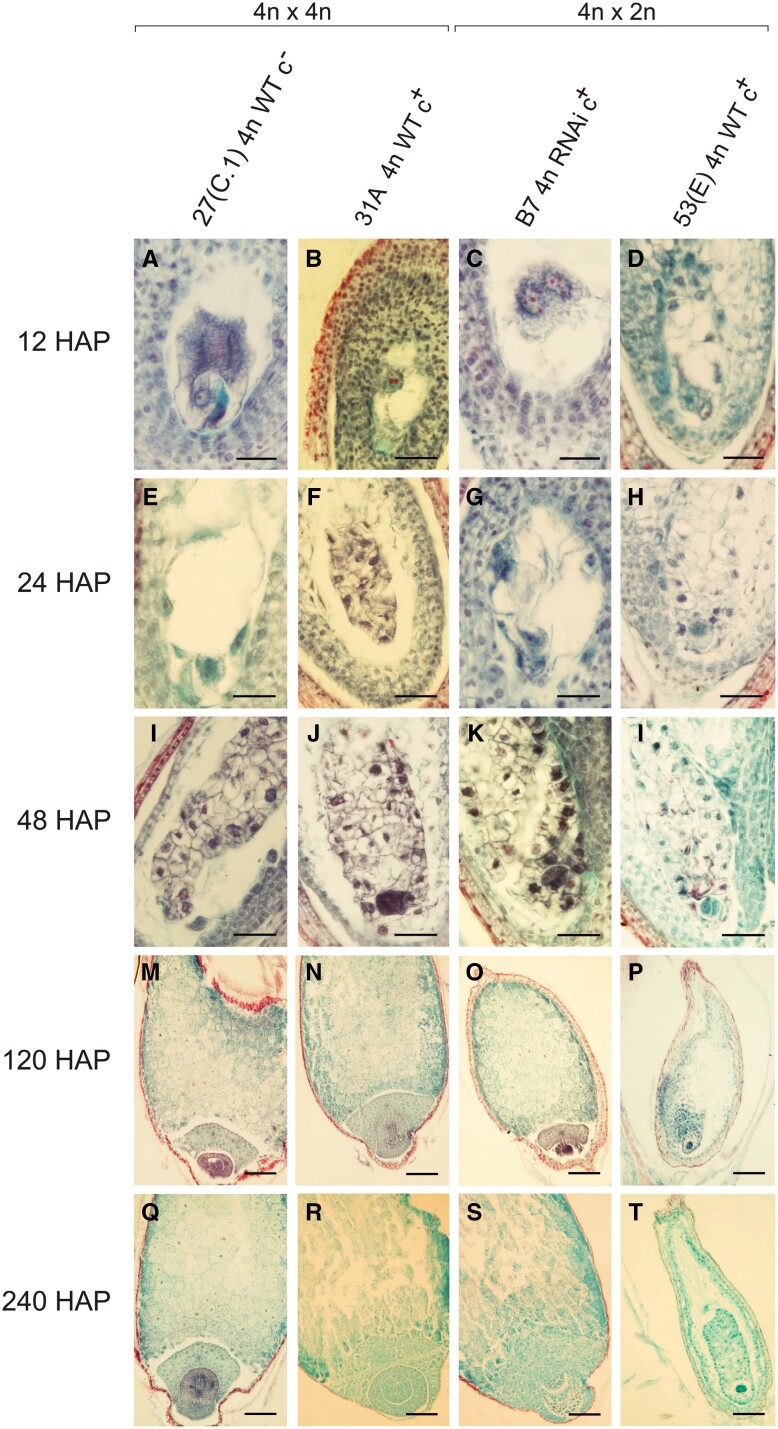
Histological analysis of developing seeds from WT and RNAi transgenic plants after homo- and interploidy crosses at 12, 24, 48, 120, and 240 hours after pollination (HAP). (A, E, I, M, Q) Longitudinal sections of developing seeds obtained by open pollination of the WT sexual tetraploid plant 27(C.1) 4*n* WT c^–^ with tetraploid pollen donors (balanced endosperm). (B, F, J, N, R) Developing seeds form natural apomictic tetraploid plant 31A 4*n* WT c^+^ derived from open pollination with tetraploid sexual plants (natural maternal excess endosperm). (C, G, K, O, S) Developing seed from the tetraploid sexual RNAi-inactivated PsORC3 RNAi-inactivated plant B7 4*n* RNAi c^+^ crossed with diploid pollen donors (induced maternal excess endosperm). (D, H, L, P, T) Developing seeds from the natural tetraploid sexual plant 53(E) 4*n* WT c^+^, crossed with diploid genotypes (induced maternal excess endosperm). Scale bar=30 µm in A–D, 50 µm in E–L, and 150 µm in (M–T).

In order to distinguish the developmental stage at which unbalanced endosperms arrested in sexual genotypes and correlate it to temporal regulation of *PsORC3* expression, comparative histological analyses were undertaken between RNAi transgenics and WT genotypes, either apomictic or sexual. A preliminary study of the female gametophyte development was carried out to investigate the possible causes of the lower seed setting in interploidy crosses involving the PsORC3 RNAi lines (2–5 seeds) compared with those involving PsORC3c^–^ WT mother plants (6–28 seeds, Table 2). Both PsORC3 RNAi lines and their corresponding WT controls showed well-differentiated megaspore mother cells (MMCs) in the nucellar tissues of the ovule primordium (Supplementary Fig. S9A, F). However, while in the WT plants the MMCs underwent regular meiosis and developed *Polygonum*-type embryo sacs (Supplementary Fig. S9B–E), in PsORC3 RNAi lines, abnormalities frequently occurred as degeneration of one or both dyads at first meiotic division, and of the whole tetrad at second meiotic division (Supplementary Fig. S9H–J). On average, only 40% of embryo sacs with a regular eight-nucleated *Polygonum*-type structure were observed in the PsORC3 RNAi lines (Supplementary Fig. S9). These alterations could be attributed to inactivation of genes involved in cell differentiation and development, probably caused by the long period of tissue culture necessary to regenerate the PsORC3 RNAi lines. Similar sterility problems were also detected in regenerated plants devoid of the PsORC3 RNAi cassette (not shown). Then, the development of the few endosperms derived from interploidy crosses involving *PsORC3*-inactivated plants, together with their related WTs, as female parents, and a pool of diploid genotypes as pollen donors, was followed from 12 to 240 HAP (Fig. 4). The comparison of endosperm development in interploidy crosses of both B7 4*n* RNAi c^+^ and its related wild type 53(G) 4*n* WT c, allowed us to verify the effect of down-regulation of *PsORC3b* and *c* isoforms in near isogenic lines, where phenotype reversion (overcoming of triploid block) was observed in plants subjected to interference. On the other hand, since co-transformed plant 1E 4n RNAi c^–^ and its control 39(G) 4*n* WT c^–^ carried the null allele for *PsORC3c*, little information was expected from this material regarding the effect of interference with *PsORC3* on development of maternal excess endosperm. Therefore, these latter crosses were not taken into consideration in this analysis. WT sexual and apomictic tetraploid individuals [27(C.1) 4*n* WT c^–^ and 31A 4*n* WT c^+^, respectively] open pollinated with tetraploid males of the same phenotype were also included as controls. Moreover, the relative abundance of immature seeds in each development stage (from fertilization to endosperm and embryo full development) recorded at 12, 24, and 48 HAP was used as a parameter to compare the timing of endosperm differentiation among different crosses (Supplementary Fig. S10). Overall, endosperm development preceded embryo formation in all cross combinations. At 12 HAP, all endosperms were in early development stages ranging from polar nuclei fertilization (Fig. 4B), late anaphase (Fig. 4A) and telophase (Fig. 4C) of the first mitotic division, to early syncytial stage (Fig. 4D), in both controls and RNAi lines. At 24 HAP, endosperms of 31A 4*n* WT c^+^ and 53(E) 4*n* WT c^+^ were the first to advance to the initial cellularization stage (Fig. 4F, H), whereas most of those developing on 27 (C.1) 4*n* WT c^–^ and B7 4*n* RNAi c^+^ were still at the early mitotic stages (Fig. 4E, G). Probably, as a first effect, the down-regulation of the *PsORC3b* and *c* isogenes delayed the development of unbalanced endosperms. At 48 HAP, most of the developing endosperms reached the cellularization stage, regardless of whether the parental contribution was balanced or not (Fig. 4I–L; Supplementary Fig. S10). At 120 HAP, early developed seeds with well-differentiated endosperm and embryo were detected in both homoploid crosses (Fig. 4M, N), and in the interploidy cross involving B7 4*n* RNAi c^+^ as seed parent (Fig. 4O). Conversely, in 53(E) 4*n* WT c^+^ developing seeds, the embryo was blocked at the pro-embryo stage and the endosperm arrested at the advanced cellularization stage (Fig. 4P). The same scenario occurred at 240 HAP, as no evident difference in seed development was observed among the various crosses (Fig. 4Q–S), except for 53(E) 4*n* WT c^+^ in which seed development remained blocked (Fig. 4T). Even further, the seeds from this last cross underwent a complete degeneration of their components a few days later (not shown). In summary, unbalanced endosperms evolved faster than balanced ones during initial stages of development, but they all reached the cellularization stage at ~48 HAP. From then on, while balanced endosperms, as well as unbalanced ones from apomictic genotypes, developed further to occupy the largest part of the caryopsis, the unbalanced endosperms derived from the *PsORC3c*-holding mother plant, arrested their development at the late cellularization stage. The unbalanced endosperms from PsORC3 RNAi plants evolved in a way similar to those observed in sexual WT balanced endosperms in early development phases but, more interestingly, they succeeded in overcoming the block at the end of cellularization to evolve into a fully developed seed.

In order to investigate whether the arrest observed in the development of the maternal excess endosperms affected the proliferative or the endoreduplicative programs, the development of seeds carrying balanced or maternal excess endosperms was compared, with the consideration of additional time points between 48 and 120 HAP in homoploid and interploidy crosses involving the same not permissive (i.e. *PsORC3c*-holding) tetraploid seed parent. Histological examinations, starting from the end of cellularization (48 HAP) to 72, 96, and 120 HAP, showed that balanced endosperm increased the number of cells to cover most of the ovule capacity and the embryo evolved from the pro-embryo/transition stage to coleoptilar/L1 late stage (Armenta-Medina *et al.*, 2021) (Supplementary Fig. S11A–D). Conversely, maternal excess endosperm maintained both the cellularized and the pro-embryo structures unchanged until 120 HAP (Supplementary Fig. S11E–H), indicating that the proliferative mitotic phase was either absent or arrested. The ploidy assay of nuclei in the developing endosperm at 120 HAP showed that in sexual balanced endosperm two endoreduplication cycles of the hexaploid nuclei (12C and 24C, Supplementary Fig. S12A) occurred. In contrast, only one cycle of decaploid nuclei endoreduplication (20C) barely took place in unbalanced endosperms (Supplementary Fig. S12B). This indicated that massive endoreduplication did not take place before 120 HAP in either balanced or natural apomictic maternal excess endosperms. In conclusion, the proliferative mitotic phase before endoreduplication could act as a checkpoint for formation of seeds with the correct EBN. Overexpression of *PsORC3c* and *b* acted as a ploidy sensor inhibiting the development of seeds with the incorrect EBN.

## Discussion

Here we have shown that the silencing of *ORC3* allows sexual tetraploid *P. simplex* plants to overcome the EBN barrier and set viable seeds carrying endosperms with an excess maternal genome contribution. We are aware of having obtained only two transgenic ORC3 RNAi genotypes (one from callus B and the other from callus 1) with their related data to support our conclusions. Neverthelesse, the outcome of transformation experiments serves as a preliminary indication of the possible role of *PsORC3b* and *PsORC3c* in maternal excess endosperm development. More robust evidence is provided from the comparison among independent interploidy crosses, in which crosses involving tetraploid *PsORC3c*^*+*^ as mother plants have remained infertile whereas those from *PsORC3c*^*-*^ have yielded viable seeds. Therefore, we suggest that *ORC3* emerges as a key determinant of endosperm development, and consequently as one of the master regulators of apomictic reproduction in this species, even if further experimental evidence is necessary to confirm these conclusions.

### PsORC3 acts as a ploidy sensor controlling correct EBN in endosperms of *P. simplex*

Seed lethality is a frequent outcome in interploidy crosses, where the cellularization phase is crucial for endosperm development (Gehring and Satyaki, 2017). Faster cellularization is a common feature in maternal excess endosperm compared with balanced endosperm. However, while in maize and rice such interploidy crosses remain infertile (Leblanc *et al.*, 2002; Sekine *et al.*, 2013), Arabidopsis generate viable, though morphologically altered, seeds (Scott *et al.*, 1998). In our material, although maternal excess endosperms developed slightly faster than balanced ones in the early phases of development, both balanced and unbalanced endosperms reached the cellularization stage 2 d after pollination. From then on, while maternal excess endosperms derived from natural apomictic or sexual plants, in which *PsORC3* was naturally down-regulated or RNAi inactivated, continued to develop, those generated by sexual WT plants, in which maternally regulated *PsORC3* was overexpressed at the onset of endoreduplication, arrested their growth. This means that the maternal genome dosage increase in the endosperm disrupts the transition from late cellularization/proliferation to endoreduplication phases and that the silencing of the maternally imprinted *PsORC3* gene is sufficient to allow the maternal excess endosperm to proceed to the endoreduplication phase, generating a fully viable seed. These results agree with the conclusion of Leblanc *et al.* (2002), who noticed that an excess of maternal genomes in maize endosperm disrupts the proliferation phase allowing premature termination of the endoreduplication phase, and have confirmed their hypothesis that maternally expressed genes related to the cell cycle may have a role in the transition between these two phases. Similar conclusions are drawn by Li and Dickinson, (2010) although the highlighted relationships between cell cycle-related and differentially expressed genes in interploidy crosses seems unclear in their study.

Even though imprinting mediated by the Polycomb Repressive Complex 2 (Kradolfer *et al.*, 2013) is the most accepted mechanism, more recent alternative processes for parent-of-origin expression have been proposed (Batista and Khöler, 2020). Among these, a more general model deals with the hypothesis that differential parental contribution to the developing seed is likely to be due to a difference in gene products, rather than to the ploidy level of the central cell compared with the pollen cell nucleus (Birchler, 2014). The product of any dosage-sensitive regulator may be pre-accumulated mRNA, proteins, or even a product related to chromosome imprinting. In any case, the complete silencing of parent-specific alleles is not required (Dilkes and Comai, 2004). Among regulatory genes involved in seed development, those encoding subunits of multiprotein complexes are the most prone to be sensitive to variation in dosage between parents (Veitia, 2002). In fact, any variation in stoichiometric balance among proteins involved in a coordinated interaction network induces detrimental phenotypes and a reduction in fitness (Veitia and Birchler, 2022). Moreover, at low concentration, the subunits acting as bridge in the multiprotein complex limit the amount of functional whole complex (Veitia 2002; Veitia *et al.*, 2008). Analysis of subunit inter-relationships within the ORC complex shows that the ORC3 subunit plays a central role in the complex assembly and maintenance in maize (Witmer *et al.*, 2003) and in Arabidopsis (Masuda *et al.*, 2004), thereby allowing the pre-RC machinery to function properly. In *P. simplex*, although the regulator *PsORC3c* is subjected to complete (binary) imprinting in interploidy crosses, its functional effector *PsORCb* is differentially expressed in developing seeds, depending on whether *PsORC3c* is contributed by the maternal or paternal parent. Therefore, *PsORC3b* might control the formation of maternal excess endosperm in sexual *P. simplex* by an indirect gene dosage effect mediated by the maternally imprinted regulator *PsORC3c.*

### ORC function might be dispensable in the endoreduplication program of endosperm in *P. simplex*

ORC proteins, as well as other components of the pre-RC, are major players in endosperm development (Sabelli and Larkins, 2009). In particular, the ORC complex is likely to be involved in steps in which DNA duplication is required, for example cell multiplication (first acytokinetic mitosis dealing with the syncytium formation and subsequent cell proliferation at the end of the cellularization stage) and endoreduplication (the stage following cell proliferation where, in cereals, DNA duplication takes place without subsequent nuclear division resulting in polyploidization of the endosperm cells). Although most of the maize mutants defective for endosperm development reveal anomalies in both cell proliferation and endoreduplication, in *dek* mutants only endoreduplication is affected, indicating that this DNA amplification program might be regulated by a specific mechanism (Kowles *et al.*, 1992). More recently, it has been reported that the transition from endosperm cell proliferation to endoreduplication is regulated by a decrease in cyclin-dependent kinases (CDKs) and an increase in the Retino Blastoma Relative (RBR) pathway (Gutierrez *et al.*, 2014), suggesting that the ORC contribution might differ in the two replicating programs. Moreover, in the endoreduplication program of leaf cells, the expression of both *ORC3* and *ORC4* is dispensable (Barkla *et al.*, 2018). Finally, based on the observation that cells of the salivary glands of *Drosophila* endoreduplicate without ORC1 (Park and Asano, 2008), and that all subunits are required for the correct function of the ORC (Chesnokov *et al.*, 2001), Asano (2009) hypothesizes that the whole ORC complex might be dispensable for endoreduplication. Rather, the ORC would be replaced by another complex able to recruit CDC6, the *Drosophila* homolog of CDT1, and the MCM complex to assemble an alternative pre-RC complex exclusively devoted to the endoreduplication program. This specificity might be related to the differences on the physiological finalities of the two replicative programs; in the case of cell proliferation, the aim of DNA duplication is to generate two identical cells that conserve the intact chromosomes of the mother cell, while the endoreduplication program deals with the amplification of gene copy number to support increased protein synthesis (Edgar and Orr-Weaver, 2001). From this perspective, endoreduplicated cells are terminally differentiated and committed to programmed cell death. Therefore, the regulatory check on DNA integrity provided by the ORC can be bypassed by the simpler regulation governed by CDC6 (Asano, 2009). Similarly, the ORC may be dispensable in the endosperm endoreduplication program of *P. simplex* apomictic seeds; rather, the overexpression of genes coding for at least some ORC subunits might have acquired the new function of ploidy sensor.

### The model of *PsORC3* isogene interplay in sexual and apomictic endosperm development in *P. simplex*

**Fig. 5.**
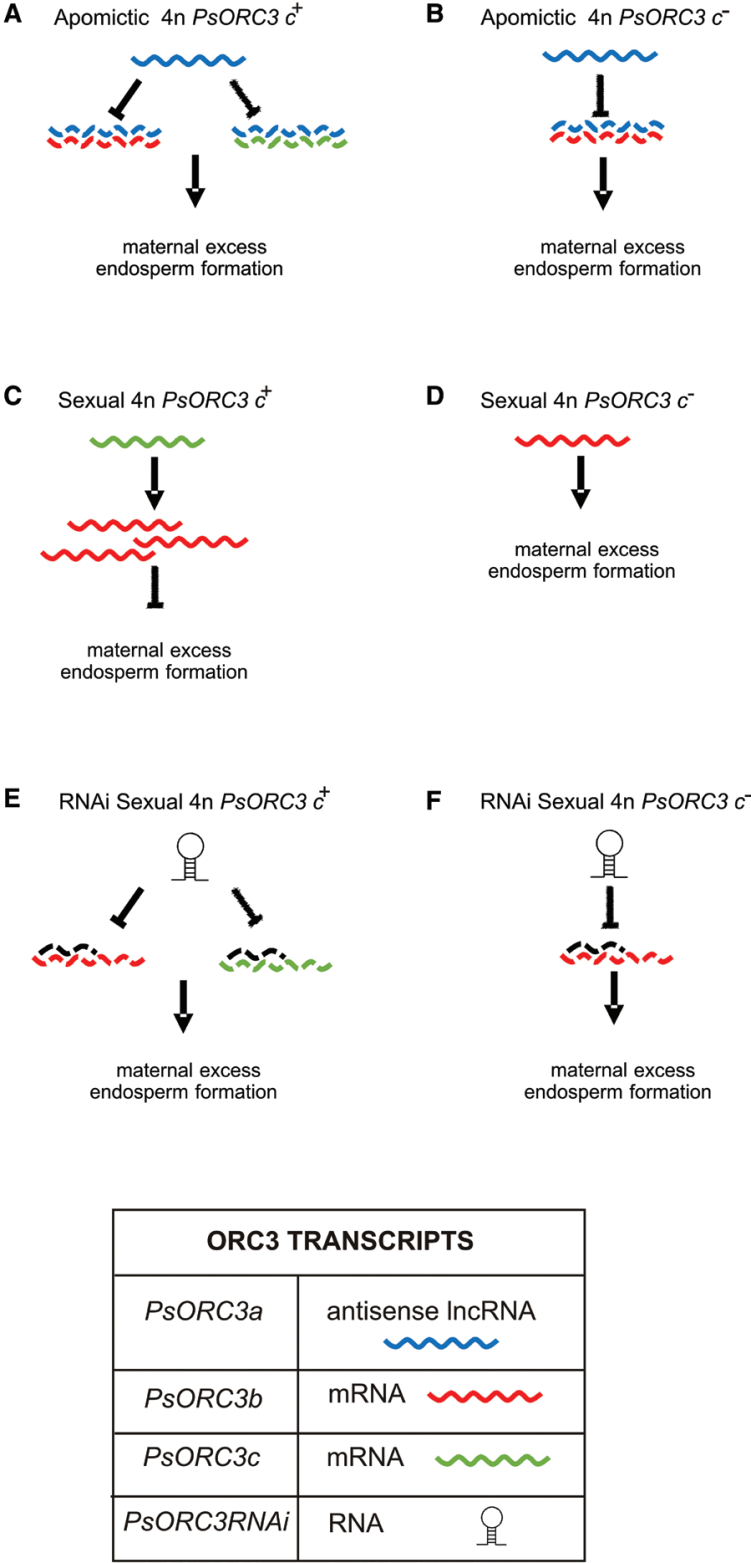
Model of maternal excess endosperm development controlled by *PsORC3* in *P. simplex*. (A, B) In apomictic plants, the presence of *PsORC3a* lncRNA in antisense orientation inhibits both *PsORC3c* (when present) and *PsORC3b* expression. This fact always allows the formation of viable seeds with maternal excess endosperm. (C, D) In interploidy control crosses, if the sexual tetraploid mother plants harbor the *PsORC3c* isogene (C), *PsORC3b* is up-regulated and no maternal excess endosperm is produced, whereas in mother plants null for *PsORC3c*, *PsORC3b* is maintained at its basal expression and the formation of triploid seeds with maternal excess endosperm is allowed (D). (E, F) In interploidy crosses in which ORC3 RNAi plants are used as seed parents, the expression of RNAi transcript mimics the action of *PsORC3a* in natural apomicts (by silencing both *PsORCb* and *c*); in both cases of *PsORC3* genotype configuration, generation of triploid seeds with maternal excess endosperm is allowed.

By means of an RNAi-based approach, we have been able to propose a model for the interplay and hierarchy among the three *ORC3* isogenes involved in the development of maternal excess endosperm in *P. simplex* (Fig. 5). *PsORC3a*, which exists only in apomictic genotypes, behaves like a dominant negative regulator of its homologs *PsORC3b* and *PsORC3c*, probably by a sense–antisense-mediated mechanism. The latter two isogenes act as a sensor of unbalanced genome contribution at late stages of endosperm development (Fig. 5A, B). In sexual tetraploids, the expression of *PsORC3c* in the maternal parent of interploidy 4*n*×2*n* crosses is always associated with the up-regulation of *PsORC3b* at late stages of endosperm development and hence with seed death (Fig. 5C). Conversely, in sexual genotypes depleted of *PsORC3c* or when this isogene is contributed by the paternal parent, the steady-state levels of *PsORC3b* mRNAs are too low to arrest the development of maternal excess endosperms (Fig. 5D). Thus, RNAi of *PsORC3* recreates, in sexual plants, the conditions that in natural apomictic plants allow the formation of maternal excess endosperm (Fig. 5E, F).

The development of unbalanced endosperm is a major concern in attempts to introduce apomixis in crops (such as rice and maize) for which the EBN is required (Ozias-Akins and Conner, 2020). One proposed strategy is to rearrange the effective ratio of maternal:paternal genomes in the endosperm based on the modification of the imprinting program of the central cell, so as to ‘paternalize’ the polar nuclei. This would restore the effective EBN from maternal excess to a 2:1 ratio (Spielmann *et al.*, 2003). This concept has been taken up and further developed by Spillane *et al.* (2004), suggesting first identifying suppressors of genomic imprinting in sexual plants and then modulating their expression to develop imprinting-insensitive genotypes able to circumvent the constriction of the EBN in the endosperm. In any case, they have proposed to find these suppressors of imprinting in sexual models or in plants for which apomixis would be desirable. Overall, the present study describes for the first time an imprinted gene, *PsORC3*, which controls the development of maternal excess endosperm in a natural apomictic plant. Thus, targeting the activity of genes involved in the expression of *PsORC3* may represent an efficient and innovative route to engineer crop tolerance to EBN deviations, ultimately leading to apomixis introgression in crops.

## Supplementary data

The following supplementary data are available at *JXB* online.

Fig. S1. Inhibition of growth and regeneration of *P. simplex* calli.

Fig. S2. Construction of the transformation vector for RNAi-mediated *ORC3* gene suppression and identification of transformed plants by PCR analyses.

Fig. S3. Optimization of *P. simplex* nuclear genome transformation parameters.

Fig. S4. Plants regenerated from calli grown on the selective agent glufosinate-ammonium or hygromycin.

Fig. S5. Banding patterns resulting from PCR amplification of *P. simplex* genomic DNA with the *PsORC3c*-specific primer pair, PsORC3cfw/PsORC3crev.

Fig. S6. Restriction fragment length polymorphism (RFLP) analysis of regenerated plants.

Fig. S7. qRT–PCR transcriptional profiles of *PsORC3b* isogenes in leaves of WT [53(E) 4*n* WT c+] and RNAi (B7 4*n* RNAi c+) plant of *P. simplex*.

Fig. S8. Amplifying banding pattern of the isogene *PsORC3c* in interploid (4*n*×2*n*) and homoploid (2*n*×2*n*) control crosses in *P. simplex*.

Fig. S9. Main abnormalities detected in macrosporogenetic development of ORC3 RNAi plants of *P. simplex*.

Fig. S10. Relative abundance of seed developmental stages among developing seeds characterized by different parental genome contributions in the endosperm.

Fig. S11. Comparative histological analysis of developing seeds, characterized by different parental genome contributions in the endosperm, from the end of cellularization to the end of proliferative/onset of endoreduplication stages.

Fig. S12. Estimated ploidy level of developing balanced endosperms derived from homoploidy (4*n×*4*n*) open crosses, by Feulgen analysis in apomictic and sexual *P. simplex*.

Table S1. List of primers and PCR amplification conditions used in this study.

Table S2. Identification of a suitable selective agent for transformation of *P. simplex* calli.

erad069_suppl_Supplementary_Figures_S1-S12_Tables_S1-S2

## Data Availability

All data supporting the findings of this study are available within the paper and within its supplementary data published online.
