# Supplementary material for: ORIGIN OF RECOGNITION COMPLEX 3 controls the development of maternal excess endosperm in the Paspalum simplex agamic complex (Poaceae)
Source: J Exp Bot. 2023 Feb 22;74(10):3074–93. doi: 10.1093/jxb/erad069 (PMC10199125; doi:10.1093/jxb/erad069)
Supplement: erad069_suppl_Supplementary_Figures_S1-S12_Tables_S1-S2 [file erad069_suppl_supplementary_figures_s1-s12_tables_s1-s2.pdf]

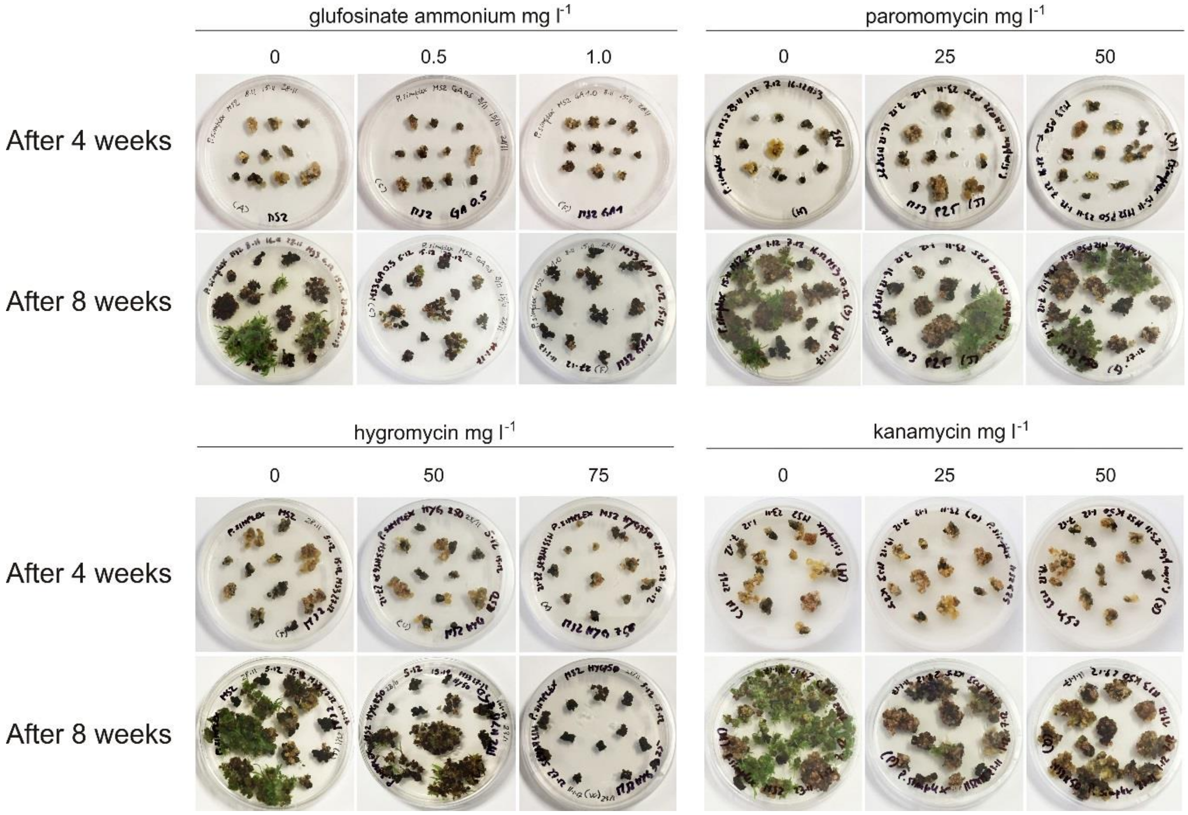

Fig. S1. Inhibition of growth and regeneration of *P. simplex* calli. Calli were grown for four weeks on MS2 regeneration medium containing glufosinate ammonium, paromomycin, hygromycin and kanamycin at the indicated concentrations. They were transferred to MS3 shoot proliferation medium with the same selection agent for four weeks. Images were taken after four (T4) and eight (T8) weeks of selection.

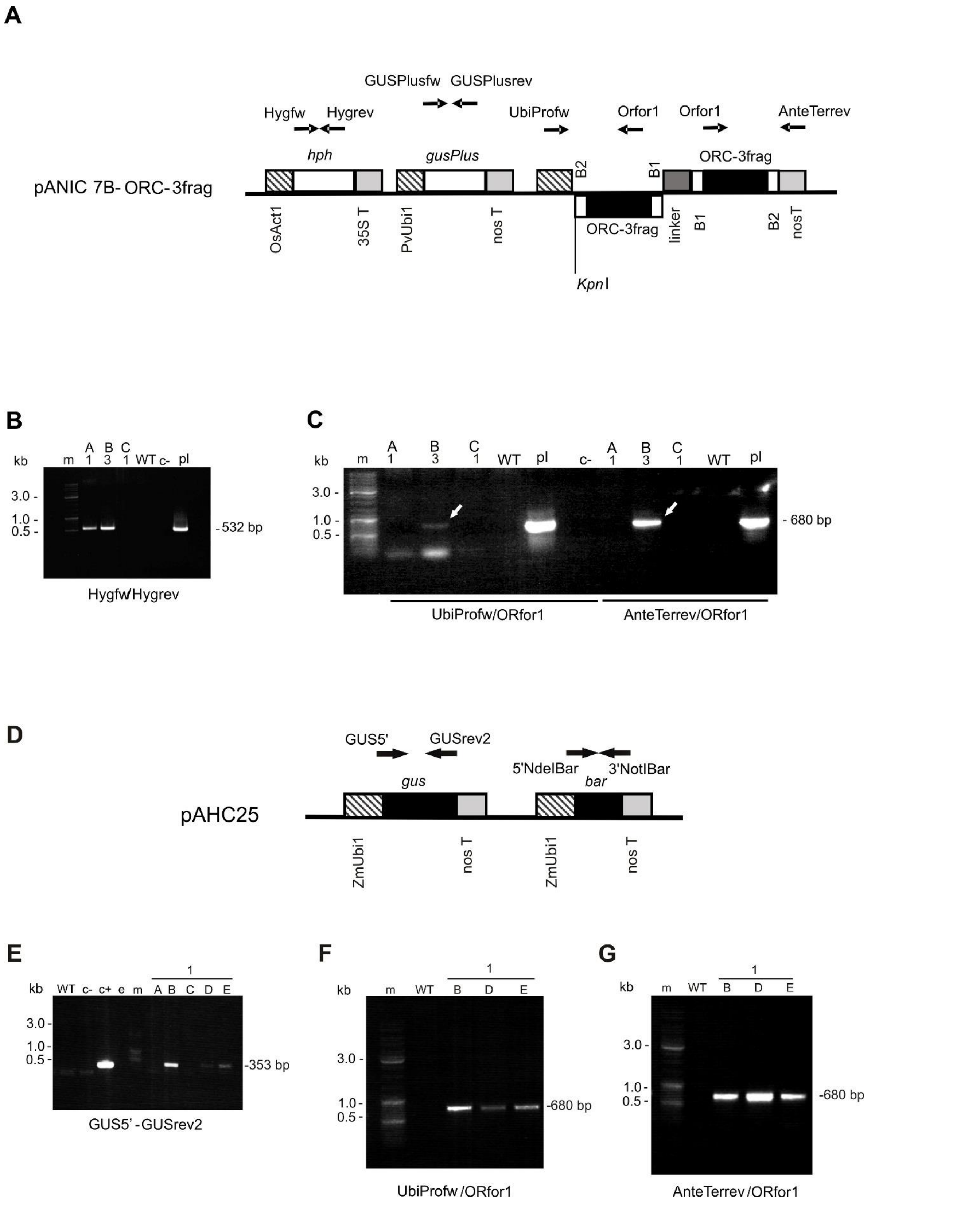

Fig. S2. Construction of the transformation vector for RNAi-mediated *ORC3* gene suppression and identification of transformed plants by PCR analyses. A) Physical map (not drawn to scale) of the transformation vector pANIC 7B-ORC-3frag. Genes above the line are transcribed from left to right, while the ORC-3frag copy below the line is transcribed in the opposite direction. The unique restriction

site of *Kpn*I in the plasmid DNA is shown. B-C) PCR amplification of three hygromycin-resistant plants (A1, B3, and C1) with primers specific for *hph*, the hygromycin resistance gene (B), and with the two primer pairs, UbiProfw/ORfor1 and AnteTerrev/ORfor1, that amplify portions of the first and second ORC-3frag copy, respectively (C). Wild-type (WT) *P. simplex* DNA and a sample without DNA (c-) were used as negative controls, while plasmid pANIC 7B-ORC-3frag (pl) as positive control. D) Physical map (not drawn to scale) of the co-transformation vector pAHC25 conferring resistance to glufosinate ammonium. PCR analyses of five co-transformed plants from callus 1 (1A-E) amplified with the primer pair specific for the GUS gene harbored by plasmid pAHC25 (E) and of 1B, 1D and 1E plants amplified with the *PsORC3* specific primers to reveal ORC-3frag insertion in both orientations (F, G). Wild-type (wt) *P. simplex* DNA and a sample without DNA (c-) were used as negative controls, while plasmid pAHC25 (c+) as positive control; e, empty lane. Abbreviations: *OsAct1* (rice actin 1 promoter and intron), *hph* (hygromycin B phosphotransferase gene), *35S T* (*35S* terminator sequence), *PvUbi1* (switchgrass polyubiquitin 1 promoter and intron), *GUSPlus* (GUSPlus™ coding region along with the rice glycine-rich protein signal peptide sequence), *nos T* (*Agrobacterium tumefaciens* nopaline synthase terminator sequence), *ZmUbi1* (maize ubiquitin 1 promoter, exon and intron), B1 and B2 (attB1 and attB2 sites), *ORC-3frag* (*ORC3* gene portion), *linker* (an *Escherichia coli*-derived *gusA* linker sequence), *gus* (β-glucuronidase gene), *bar* (bialaphos acetyltransferase gene), conferring resistance to bialaphos that in microorganisms and plants is converted to phosphinothricin (also dubbed glufosinate). The annealing positions of primers Hygfw, Hygrev, UbiProfw, ORfor1, GUSPlusfw, GUSPlusrev, AnteTerrev, GUS5'-GUSrev2, and 5'NdeIBar/3'NotIBar are shown. The molecular mass of the amplification products is indicated in bp; m (molecular weight marker).

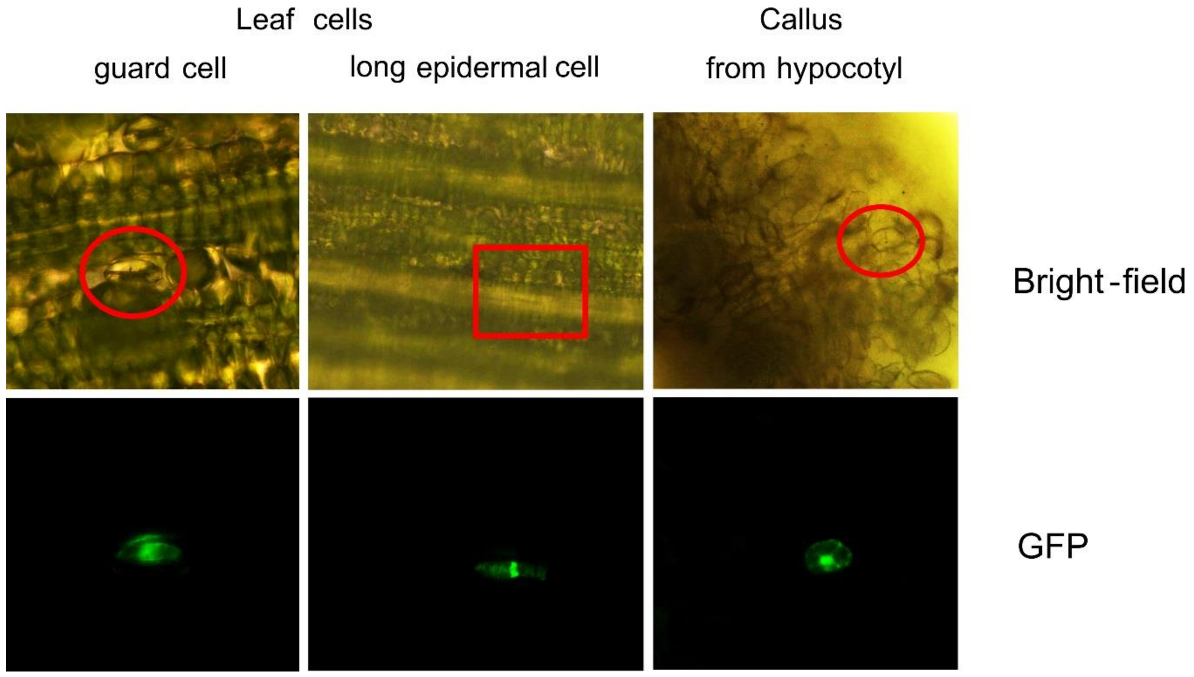

Fig. S3. Optimization of *P. simplex* nuclear genome transformation parameters. Images in the lower part of the figure represent examples of GFP visualization in cells of transiently transformed *P. simplex* leaf

(stomatal guard cell and long epidermal cell) and callus. In the upper part of the figure the corresponding bright-field images are shown.

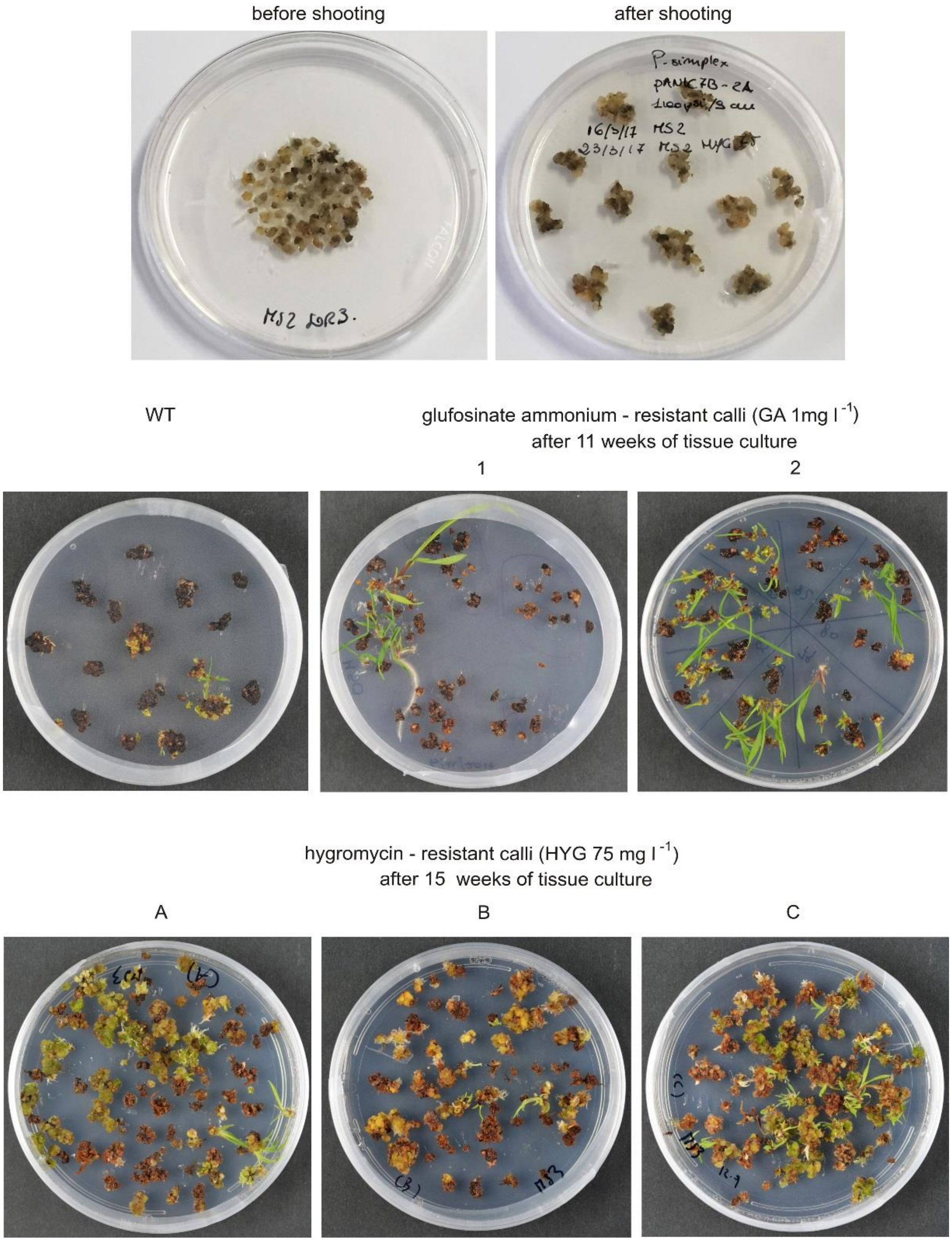

Fig. S4. Plants regenerated from calli grown on the selective agent glufosinate ammonium or hygromycin. Top) An example of *P. simplex* calli before (left) and after shooting, in MS2 supplemented with hygromycin 75 mg l^{-1} (right). Middle) Shoots from glufosinate ammonium resistant calli 1 and 2. Bottom) Shoots regenerating from three hygromycin-resistant calli (A, B, C).

A

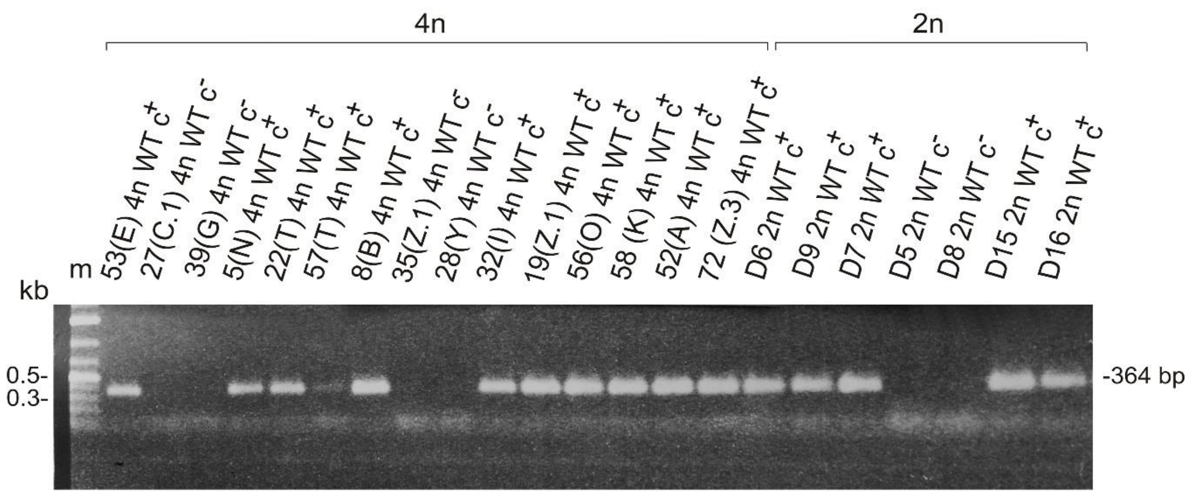

B

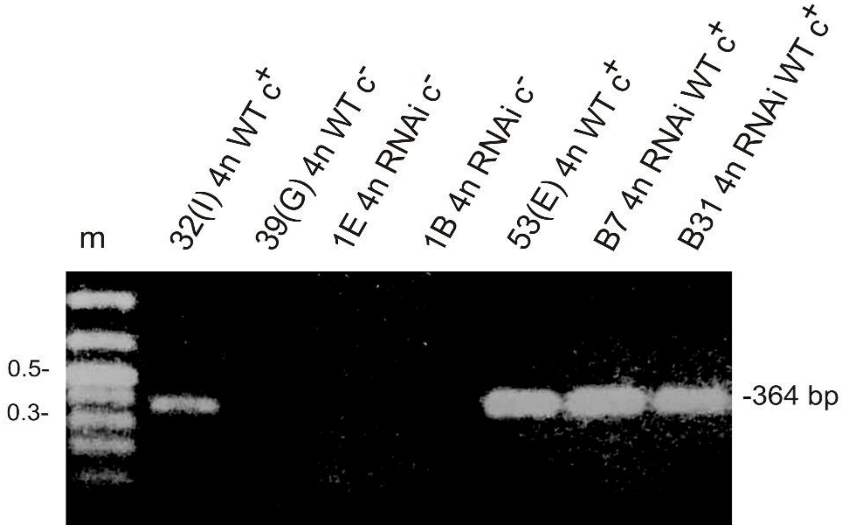

Fig. S5. Banding patterns resulting from PCR amplification of *P. simplex* genomic DNA with the *PsORC3c*-specific primer pair, PsORC3cfw/PsORC3crev. A) PCR amplification of tetraploid (4n) and diploid (2n) genotypes of *P. simplex*. B) PCR amplification of co-transformed plants (1E 4n RNAi c^- and 1B 4n RNAi c^-) and transformed plants (B7 4n RNAi c^+ and B31 4n RNAi c^+), along with their seed donor control plants (39(G) 4n WT c^- and 53(E) 4n WT c^+, respectively); the DNA of the plant 32(I) 4n WT c^+ was used as positive control. m indicates molecular weight standard.

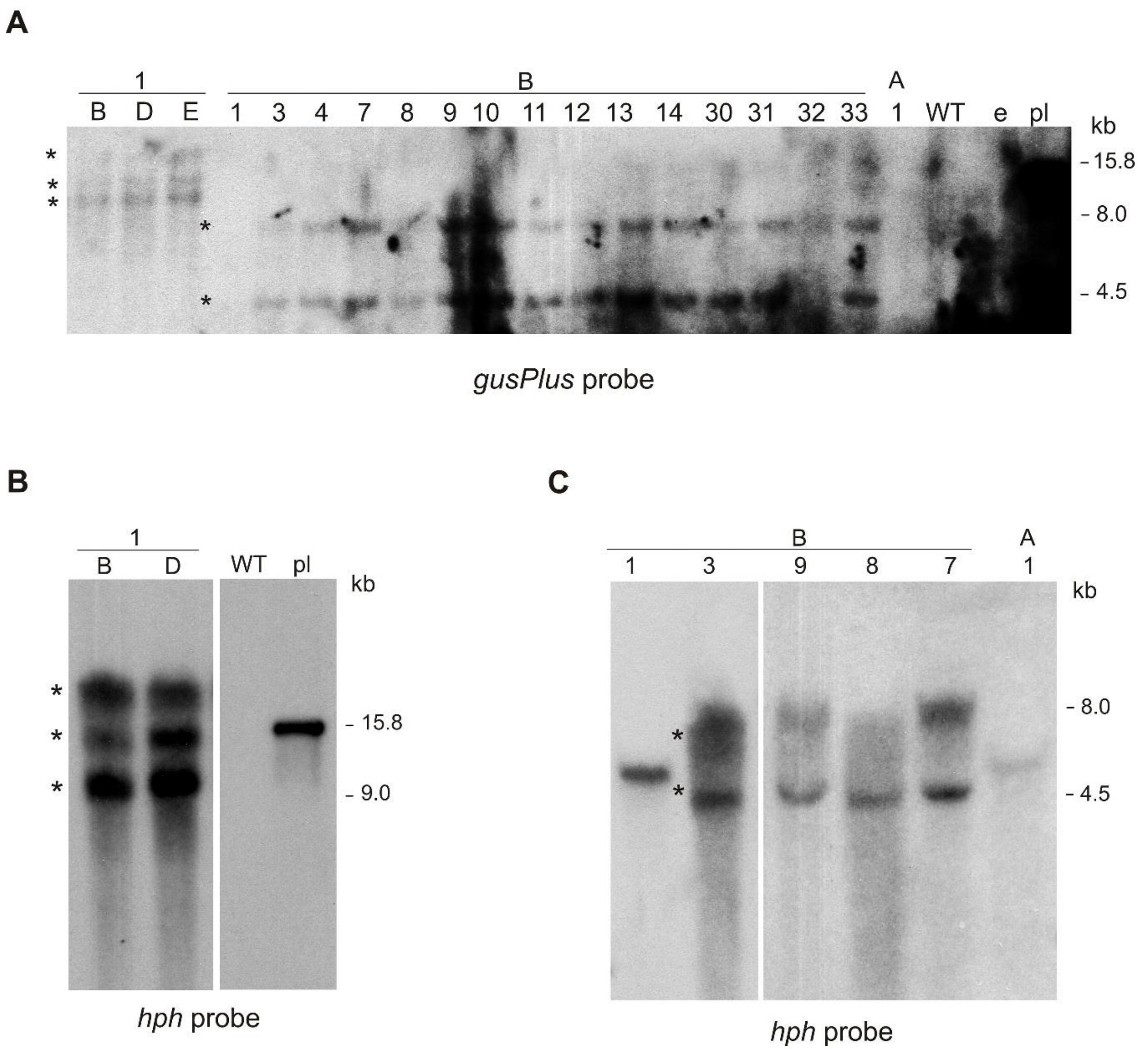

Fig. S6. Restriction fragment length polymorphism (RFLP) analysis of regenerated plants. a) Hybridizing banding pattern obtained with the *gusPlus* probe on *Kpn*I-digested DNA of three plants (B, D, and E) from callus 1, 15 plants from callus B and one from callus A. b) Hybridizing banding pattern obtained with the hygromycin B phosphotransferase (*hph*) probe on *Kpn*I-digested DNA of two plants (B and D) from callus 1, five plants from callus B and one plant from callus A (c). Abbreviations: WT (wild type), e (empty lane), pl (115 pg of *Kpn*I-restricted pANIC 7B-ORC-3frag vector, length 15,834 bp). Asterisks indicate the RFLP bands due to the transgenic multiple insertion events.

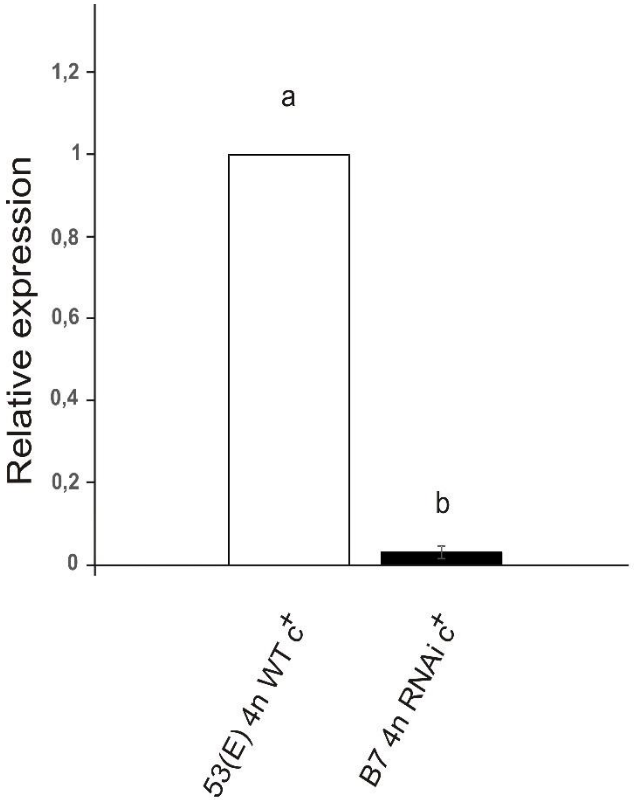

Fig. S7. qRT-PCR transcriptional profiles of *PsORC3b* isogenes in leaves of WT (53(E) 4n WT c^+) and RNAi (B7 4n RNAi c^+) interfered plant of *P. simplex*. Different letters on the top of the histograms indicate significant differences for the expression for $p \leq 0.005$. Bars on the top of histograms indicate the SEs (SE on WT is close to 0). The relative expression level refers to the expression value of the *PsORC3b* isogene of the sample 53(E) 4n WT c^+ arbitrarily set to 1.

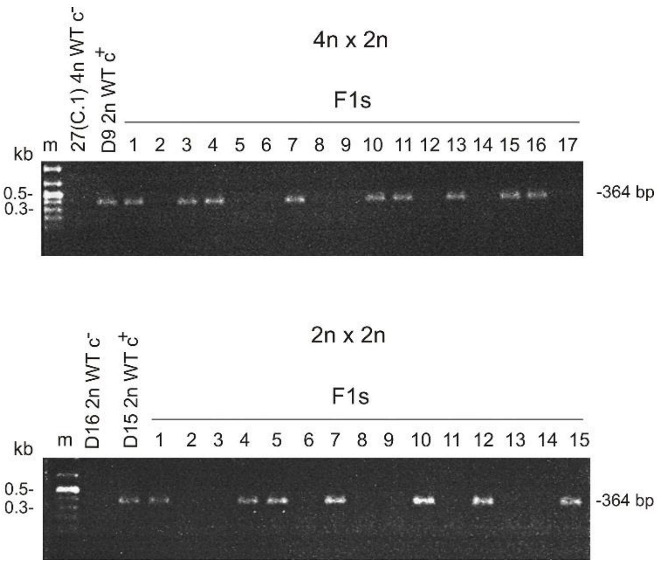

Fig. S8. Amplifying banding pattern of the isogene *PsORC3c* in interploidy (4n x 2n) and homoploid (2n x 2n) control crosses in *P. simplex*. Parent lines are reported from left (seed parent first) and numbers indicate single F1 plants. m indicates molecular weight standard.

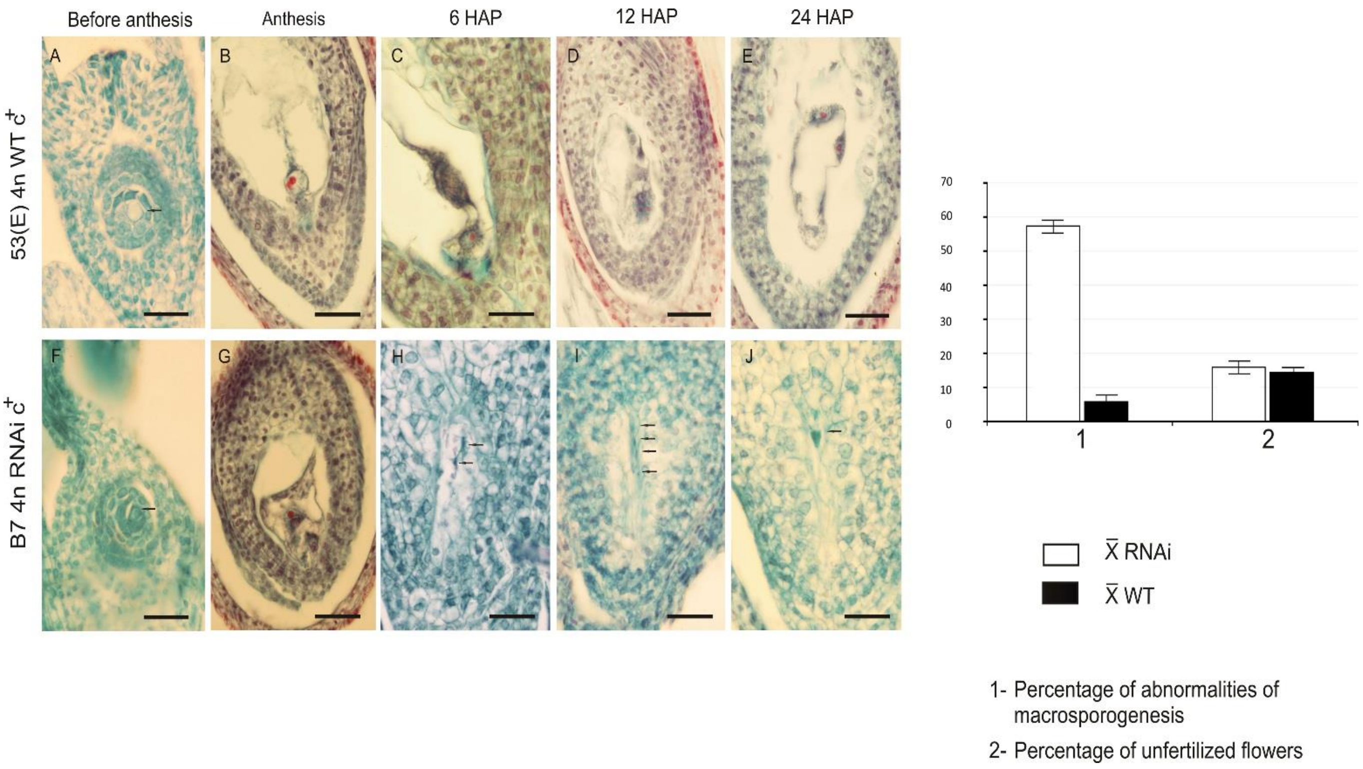

Fig. S9. Main abnormalities detected in macrosporogenetic development of ORC3 RNAi plants of *P. simplex*. Longitudinal sections of open-pollinated ovules from 53(E) 4n WT c$^+$ control plant (A-E) and from the corresponding transgenic line, B7 4n RNAi c$^+$ (F-J). Megaspore mother cell (arrow) before anthesis (A, F). Advanced seed development in control plant: B) double fertilization, C) early endosperm mitotic divisions, D) endosperm in advanced syncytial stage. Abnormalities in B7 4n RNAi c$^+$: degenerated dyads after first meiotic division (arrows, H); a degenerated tetrad after second meiotic division (arrows, I); a single degenerated cell from a delayed dyad (arrow, J). Scale bar = 30 µm (a-g); 50 µm (H-J). The graph, on the right side of the longitudinal sections, shows the percentage of (1) macrosporogenesis abnormalities and (2) unfertilized flowers, calculated as a mean of the values obtained in open-pollinated PsORC3 RNAi lines (B7 4n RNAi c$^+$ and 1E 4n RNAi c$^-$) or in their corresponding open-pollinated recipient genotypes (53(E) 4n WT c$^+$ and 39(G) 4n WT c$^-$, respectively).

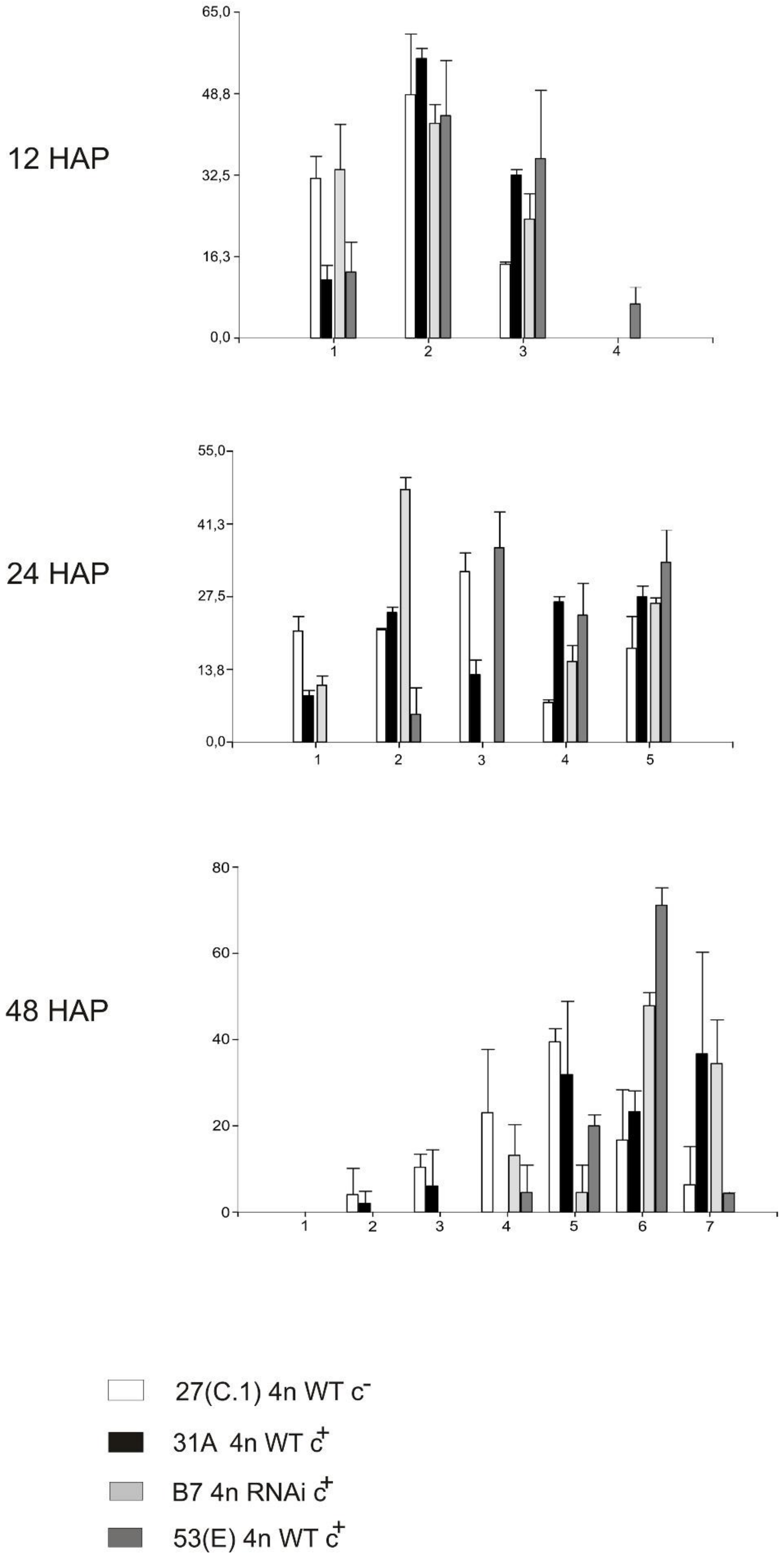

Fig. S10. Relative abundance of seed developmental stages among developing seeds characterized by different parental genome contribution in the endosperm. Sexual 27 (C.1) 4n WT c^{-} and apomictic 31A 4n WT c^{+} refer to homoploidy 4n x 4n crosses, with 2m:1p balanced genome ratio in the endosperm in

the former case and 4m:1p unbalanced genome ratio in the endosperm in the latter. B7 4n RNAi c^+ and 53(E) 4n WT c^+ refer to interploidy 4n x 2n crosses with 4m:1p unbalanced genome ratio in the endosperm. Forty florets of 27 (C.1) 4n WT c^-, 31A 4n WT c^+, 53(E) 4n WT c^+, and twenty of B7 4n RNAi c^+, for each HAP, were collected from two different inflorescences, and used to prepare the paraffin sections. In Y axis the percentages of detected phases are reported. In X-axis the developmental stages are indicated as follows: 1, fertilization; 2, first mitotic divisions; 3, syncytium with more than 20 nuclei; 4, nuclei localized at periphery of the embryo sac; 5, cellularization (embryo with more than 4 nuclei); 6, cellularization (globular embryo); 7, endosperm and embryo well developed. At 12 HAP, 31A 4n WT c^+ and 53(E) 4n WT c^+ showed the lowest percentage of developing endosperms with a single nucleus (stage 1) and the highest percentages of 53(E) 4n WT c^+ endosperms at the stage of syncytia with more than 20 nuclei, or even at later stages, (stages 3 and 4) thus witnessing a faster development of natural unbalanced endosperms at this early phase. At 24 HAP, about 30% of endosperms of 31A 4n WT c^+ and 53(E) 4n WT c^+ advanced to the early cellularization stage, whereas only 15% of endosperms of 27(C.1) 4n WT c^- remained in the same stage (stage 5). At the same time, most of the B7 4n RNAi c^+ endosperms analyzed (50%) were still at first mitotic division stage (stage 2). At 48 HAP, most of the developing endosperms reached the cellularization stage, (stages 5 and 6).

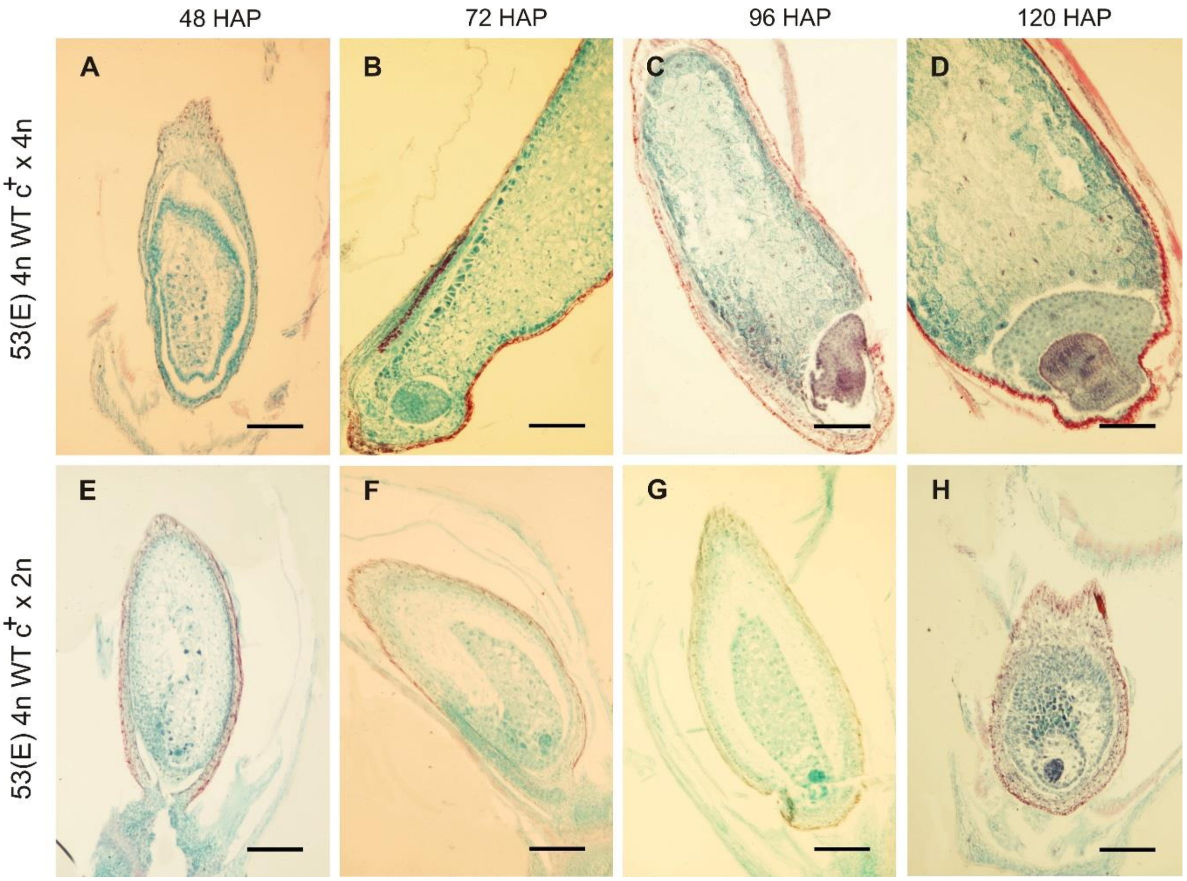

Fig. S11. Comparative histological analysis of developing seeds, characterized by different parental genome contribution in the endosperm, from the end of cellularization to end of proliferative/onset of endoreduplication stages. Longitudinal sections of developing seeds derived from 53(E) 4n WT c^+, cross-pollinated with: (A-D), tetraploid genotypes (balanced endosperm) and (E-H) diploid genotypes (maternal excess endosperm). HAP = Hours After Pollination. Bar = 150 µm.

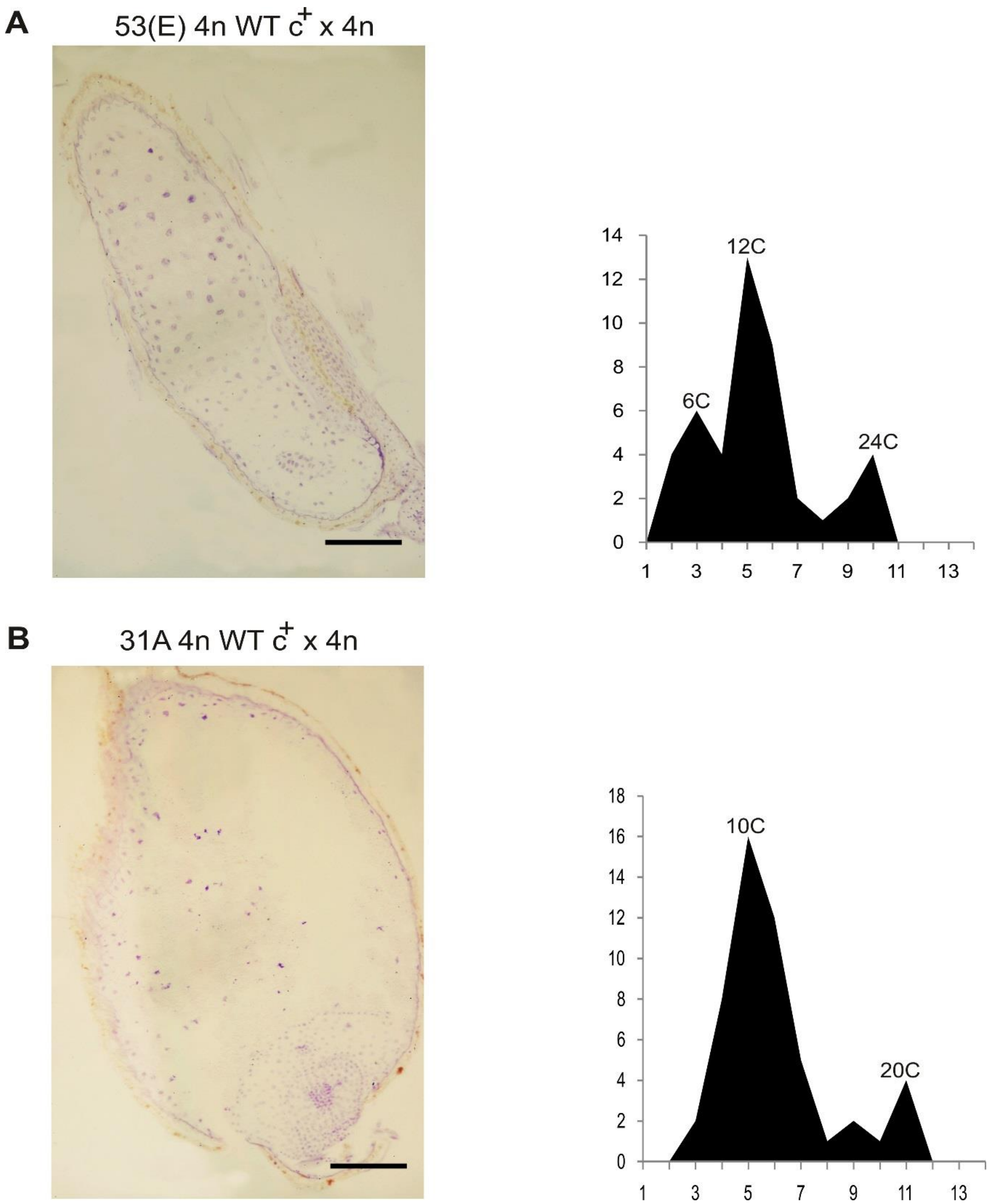

Fig. S12. Estimated ploidy level of developing balanced endosperms derived from homoploid (4n x 4n) open crosses, by Feulgen analysis in apomictic and sexual *P. simplex*. Longitudinal sections at 120 HAP of sexual (53(E) 4n WT c^{+} x 4n) and apomictic (31A 4n WT c^{+} x 4n) early seeds stained with Feulgen (left), and estimated DNA content based on nuclei Feulgen adsorption (right). Scale bar = 150 µ

Table S1. List of primers and PCR amplification conditions used in this study

Gene	Primer name	Sequence 5’→3’	Amplicon size (bp)	Purpose	PCR conditions
Hph	Hygfw	ATGAAAAAGCCTGAACTCACC	532	Cloning/amplifying the *hph* gene for transformation analysis	95°C for 5 min (1 cycle); 95°C for 30s, 60°C for 30s, 72°C for 60s (30 cycles); final extension 72°C for 7 min
	Hygrev	CCAAAGCATCAGCTCATCGA			
PsORC3	ORfor1	GGTTCAGACCTCACAGCCATA	614	Cloning a PsORC3-3frag fragment for RNAi vector construction	95°C for 3 min (1 cycle); 95°C for 30s, 62°C for 30s, 72°C for 45s (30 cycles); final extension 72°C for 20 min
	ORrev1	CAGCACATCACCGGAACATTG			
	UbiProfw	GGTGTTACTTCTGCAGGTCG	680	Amplifying a *PsORC3*-3frag fragment in antisense orientation for transformation analysis	95°C for 5 min (1 cycle); 95°C for 30s, 60°C for 30s, 72°C for 60s (30 cycles); final extension 72°C for 7 min
	ORfor1	GGTTCAGACCTCACAGCCATA			
	AnteTerrev	TCGGATCCACTAGTAACGGC	680	Amplifying a PsORC3-3frag fragment in sense orientation for transformation analysis	95°C for 5 min (1 cycle); 95°C for 30s, 60°C for 30s, 72°C for 60s (30 cycles); final extension 72°C for 7 min
	ORfor1	GGTTCAGACCTCACAGCCATA			
Gus	GUSPlusfw	CGACCTCAATGGCGTCTGGA	733	Cloning the *gus* probe for Southern analysis	95°C for 5 min (1 cycle); 95°C for 30s, 60°C for 30s, 72°C for 60s (30 cycles); final extension 72°C for 7 min
	GUSPlusrev	GCCTTCCTCATCCACGACCG			
	GUS5'	ATGTTACGTCCTGTAGAAACCC	353	Amplifying a *gus* fragment for transformation analysis	95°C for 5 min (1 cycle); 95°C for 30s, 58°C for 30s, 72°C for 30s (30 cycles); final extension 72°C for 7 min
	GUSrev2	GACATCGGCTTCAAATGGCGTA			
Bar	5'NdeIBar	TGTGCACCATATGAGCCCAGAACGACGC	531	Amplifying a *bar* fragment for transformation analysis	95°C for 5 min (1 cycle); 95°C for 30s, 60°C for 30s, 72°C for 30s (30 cycles); final extension 72°C for 7 min
	3'NotIBar	TGTGCACTGAGCGGCCGCTCAAATCTCGGTGACGGGCAG			
PsORC3c	PsORC3cfw	GCCTGGTCCAGACCTCATC	364	Amplifying the *PsORC3c* isogene specific fragment	94°C for 1 min (1 cycle); 94°C for 20s, 58°C for 20s, 72°C for 30s (45 cycles); final extension 72°C for 5 min
	PsORC3crev	GTGATGTCATCAACAAACTCCGT			

Table S2. Identification of a suitable selective agent for transformation of *P. simplex* calli

Selecting agent	C	GA		HYG		KN		PA	
Concentrations	-	0.5 mg l^{-1}	1.0 mg l^{-1}	50 mg l^{-1}	75 mg l^{-1}	25 mg l^{-1}	50 mg l^{-1}	25 mg l^{-1}	50 mg l^{-1}
Weight at T0 (g)	0.05 ± 0.01	0.03 ± 0.01	0.04 ± 0.02	0.05 ± 0.02	0.04 ± 0.02	0.03 ± 0.01	0.03 ± 0.01	0.03 ± 0.01	0.03 ± 0.01
Weight at T4 (g)	$0.17^{a} \pm 0.05$	$0.08^{a} \pm 0.04$	$0.06^{b} \pm 0.02$	$0.15^{a} \pm 0.08$	$0.08^{b} \pm 0.02$	$0.15^{a} \pm 0.08$	$0.17^{a} \pm 0.09$	$0.15^{a} \pm 0.10$	$0.16^{a} \pm 0.12$
Growth increase	340	266	150	300	200	500	566	500	533
Regenerating calli %	40	32	9	27	0	9	0	27	9

C, control with no selection agent added in the MS2 medium; GA, glufosinate ammonium; HYG, hygromycin; KN, kanamycin; PA, paromomycin. T0, initial calli weight; T4, calli weight after 4 weeks. T0 and T4 are mean values ± standard errors, calculated on a sample of 44 calli for each medium. Growth increase is expressed as the ratio T4/T0 %. Regenerating calli, the percentage of calli with regenerated shoots. Values labelled with different letters differ for $P < 0.05$ according to analysis of variance (ANOVA)
